# Quantitative analysis of China’s selenium-enriched policy texts: Based on a three-dimensional framework of “theme–instrument–effectiveness”

**DOI:** 10.1371/journal.pone.0360381

**Published:** 2026-10-13

**Authors:** Pengfei Xu, Xiongchao Xu, Qing Wang, Wenrong Ye, Jianli Luo

**Affiliations:** 1 The 11th Geological Brigade of Zhejiang Province, Wenzhou, Zhejiang, China; 2 Wenzhou University, Wenzhou, Zhejiang, China; Kintampo Health Research Centre, GHANA

## Abstract

Selenium, as an essential trace element for human health, plays a critical role in antioxidant defense, immune regulation, and disease prevention. With approximately 72% of China’s soil area being selenium-deficient, developing the selenium-enriched industry has become a strategic priority for local governments under the “Healthy China” and Rural Revitalization initiatives. While existing research has extensively explored selenium’s scientific mechanisms and industrial development pathways, a critical research gap remains in the systematic quantitative evaluation of selenium-enriched policy texts. This study addressed this gap by conducting a comprehensive quantitative analysis of 42 selenium-enriched policy documents issued in China from 2009 to 2025. Employing a three-dimensional analytical framework of “policy theme–policy instrument–policy effectiveness” integrated with Latent Dirichlet Allocation (LDA) topic modeling, content analysis, and the Policy Modeling Consistency (PMC) index model, this study reveals three key findings: (1) Current policies heavily emphasize industrial cultivation and brand building while paying insufficient attention to long-term selenium resource management and cross-regional coordination; (2) Policy instruments predominantly rely on supply-side inputs and indirect market cultivation, with weak direct stimulation of consumer health demand; (3) While policy design demonstrates good operationality (average PMC index = 6.9), it exhibits deficiencies in long-term strategic planning and national-level top-level design. This study provides actionable insights for policymakers to strengthen national top-level design, balance short-term industrial growth with long-term resilience construction, and optimize policy tool configurations to stimulate endogenous market vitality for the high-quality development of the selenium-enriched industry.

## Introduction

Selenium, as an essential trace element for the human body, plays an irreplaceable role in antioxidant defense, immune regulation, and cancer prevention [1,2]. The geochemical distribution of selenium in soils is primarily determined by geological processes during rock weathering and mineral formation, with significant spatial heterogeneity observed both globally and within China. Selenium cycling across soil-plant-atmosphere interfaces constitutes a complex biogeochemical system that directly influences selenium bioavailability in terrestrial ecosystems [3].

Given this geochemically determined distribution pattern, it is concerning that approximately 72% of China’s soil area is selenium-deficient [4], making scientific selenium supplementation a critical public health issue. Compounding this challenge, emerging evidence indicates that selenium deficiency risk is predicted to increase under future climate change [5]. Climatic factors–-including precipitation patterns, temperature regimes, and soil moisture–-directly determine selenium concentration and mobility in soils through weathering rates, leaching processes, and organic matter decomposition. The observed global decrease in soil selenium levels over recent decades represents a climate-driven phenomenon that introduces additional complexity to policy-making, while also raising important questions about whether certain Chinese soils might become selenium-enriched through future climatic and meteorological processes.

Against this backdrop of public health concerns and climatic uncertainties, developing the selenium-enriched industry-thereby transforming resource advantages into economic benefits and health outcomes-has become a key strategy for local governments to promote high-quality development of specialized agriculture within the context of the “Healthy China” initiative and the rural revitalization strategy. This industry, spanning agricultural products, functional foods, and health supplements, delivers value across environmental stewardship, public welfare, and economic growth, aligning inherently with core sustainability principles. Such a development model, which seeks to deliver concurrent value across environmental stewardship, public welfare, and economic growth, aligns inherently with the core principles of sustainability. A robust sustainability framework is defined by its simultaneous integration of ecological, social, and economic dimensions [6]. Grounded in this holistic perspective, selenium-enriched industry policies must therefore strike a deliberate balance between environmental protection, public health improvement, and regional economic development.

Academic research on selenium-enriched topics has coalesced around two principal streams of inquiry. The first stream delves deeply into the scientific mechanisms of selenium, systematically elucidating its metabolic pathways and physiological functions [7–9]. The second stream focuses on industrial economics and development pathways. Through case studies, this line of research diagnoses prevalent challenges such as lagging brand building, short industrial chains, and insufficient technological support [4,10]. While these studies consistently emphasize the need for strengthened policy support, they have largely neglected rigorous, data-driven analysis of the policy system itself.

This study addressed this critical research gap by conducting a systematic quantitative evaluation of China’s selenium-enriched policy texts. Despite the general consensus on the importance of policy support, there remains a notable absence of investigation into how policy instrument portfolios are configured, their internal consistency, and strategic orientations. This gap is crucial because the strategic selection and combination of policy instruments are key determinants of successful implementation and ultimate policy effectiveness. Without a detailed dissection of the internal architecture of the policy system, any recommendations for optimization run the risk of remaining impressionistic generalizations.

Building upon this identified research gap, this study conducted a quantitative evaluation of 42 selenium-enriched policies issued in China. It proposed a three-dimensional analytical framework–-policy theme, policy instrument, and policy effectiveness–-to answer the following research questions:

RQ1. What are the core thematic components of the current selenium-enriched policy system? What priorities and trade-offs emerge across different dimensions, including industrial cultivation, health promotion, technological innovation, and sustainability?

RQ2. What types of policy instruments have policymakers primarily deployed? What strategic orientation and resource allocation patterns are reflected in the structural distribution of these instruments?

RQ3. How internally consistent and well-designed is the policy system? Where are the critical weaknesses in policy design located?

To address these questions, this study adopted a mixed-methods approach, integrating LDA topic modeling for theme extraction, content analysis for identifying and classifying policy instruments, and the Policy Modeling Consistency (PMC) index model for quantitatively assessing the internal consistency of representative policies.

The contributions of this study are both theoretically relevant and practically significant. At the theoretical level, it applies a cutting-edge public policy analysis framework to the emerging interdisciplinary field of selenium enrichment, thereby providing micro-level empirical evidence for understanding the design of specialized agricultural policies with composite objectives. At the practical level, the findings offer policymakers a clear “policy blueprint” and an evidence-based diagnostic tool, helping to identify both the strengths and blind spots of existing policy mixes. This, in turn, provides actionable pathways for precise policy optimization and the pursuit of synergistic effects in future initiatives. The remainder of this paper is organized as follows. Materials and methods reviews the relevant literature, outlines the research design, and details the construction of the three-dimensional analytical framework. Results and discussion presents the empirical findings. Conclusions synthesizes the core findings, discusses the theoretical and practical implications, and outlines directions for future research.

## Materials and methods

This section presents the research design, data sources, and analytical methodologies, including literature review, policy text collection procedures, the three-dimensional analytical framework, and specific techniques (LDA topic modeling, content analysis, and the Policy Modeling Consistency (PMC) index model) employed for systematic policy evaluation.

### Literature review

#### Scientific merits and development dilemmas of the selenium-enriched industry.

The biological functions of selenium constitute the scientific cornerstone underpinning the development of the selenium-enriched industry. Selenium’s essential role as a trace element for humans was first conclusively demonstrated by Schwarz and Foltz in 1957 [11]. Subsequently, in 1973, Rotruck et al. further identified selenium as an integral component of glutathione peroxidase, thereby establishing its biochemical function at the molecular level [12]. In the same year, the World Health Organization officially recognized selenium as an essential trace element for humans.

Since then, academic inquiry into selenium has deepened considerably, yielding a substantial body of research elucidating its physiological functions in antioxidant defense, immune modulation, heavy metal antagonism, and cancer prevention [1,13–15]. Within the domains of agricultural and food science, research has concentrated on the development and optimization of production technologies for selenium-enriched foods. Systematic investigations have explored the uptake, transformation, and metabolic pathways of selenium in crops and livestock. A central insight from this line of inquiry is that selenium-enriched foods, in essence, rely on biotransformation processes to convert inorganic selenium into organic selenium species, which exhibit both higher safety profiles and enhanced bioavailability. This biotransformation represents a critical juncture for achieving effective and safe selenium supplementation [7,8,16].

However, dietary selenium intake among the Chinese population generally falls below recommended levels [17]. Consequently, the development of the selenium-enriched industry and the production of selenium-enriched agricultural products have emerged as a safe and effective strategy to enhance national selenium intake. As highlighted by [18], factors such as soil selenium content, its bioavailability, and the geochemical relationship between selenium and heavy metals directly determine the usability and developmental value of selenium-enriched lands. This understanding establishes a foundational basis for the scientific planning of the selenium-enriched industry.

As the industry has evolved in practice, scholarly attention has increasingly turned to the practical dilemmas encountered during its development. A noticeable trend toward product homogenization has emerged within the current selenium-enriched food sector. Product development often lacks substantive scientific and technological support, and the industrial chain remains excessively short-factors that severely constrain the industry’s sustainable growth [19]. Wang’s study of Ankang City revealed that, despite theregion’s abundant selenium resources, persistent challenges include an underdeveloped industrial chain, insufficient deep processing capabilities, and difficulty in translating superior quality into premium pricing [4]. Empirical investigations by Cao in Shanggao County, Jiangxi Province, and by Wang in Ruzhou City, Henan Province, further corroborate that weak brand influence, a shortage of scientific and technological talent, and ambiguous standard systems represent common bottlenecks restricting the development of county-level selenium-enriched industries [10,20]. Collectively, these studies have painted a realistic picture of the selenium-enriched industry from diverse perspectives, providing rich empirical material for understanding the policy support required to foster its development.

Beyond the Chinese context, a recent narrative review systematically examined and compared selenium-related regulations and standards across multiple jurisdictions–-including the Codex Alimentarius Commission, the European Union, China, and the United States–-revealing persistent challenges such as inconsistent product determination criteria, incomplete technical guidelines, and a lack of unified labeling regulations [21]. This underscores the global relevance of harmonized regulatory frameworks and offers valuable comparative benchmarks for understanding alternative governance models in the selenium domain.

#### Bridging policy instruments and quantitative evaluation in selenium-enriched research.

To systematically analyze government intervention, policy instrument theory provides a robust analytical framework. The classic tripartite taxonomy proposed by Rothwell and Zegveld (1985)-which categorizes policy instruments into supply-side, demand-side, and environmental types-offers a solid theoretical foundation for deconstructing policy texts [22]. This classification has been widely validated across various policy domains, including innovation policy, industrial policy, and agricultural policy, demonstrating its strong applicability in analyzing resource allocation patterns and strategic orientations of government intervention. Supply-side instruments manifest as direct governmental input of resources; demand-side instruments focus on stimulating the industry from the market end; and environmental instruments aim to establish an institutional framework [23]. The strategic crux lies in the optimal combination and coordinated configuration of these instruments [24]. Although the literature provides normative models of ideal policy mixes [25], empirical research rarely offers a quantitative analysis of which policy instruments have actually been deployed to promote the development of specialized industries.

A significant methodological trend involves the application of text-based quantitative policy analysis for systematic and objective evaluation [26]. Techniques such as content analysis enable the systematic classification of policy instruments [27], while computational methods like LDA topic modeling allow for the objective identification of latent thematic priorities within policy documents [28]. Moreover, indices such as the Policy Modeling Consistency (PMC) index construct a multi-variable framework, enabling a quantitative assessment of policy consistency and design quality [29].

Although these methodologies have been applied in fields such as artificial intelligence [30] and the digital economy [31], their integrated application to the evaluation of selenium-enriched policies-which encompass the multiple objectives of health, economy, and society-remains exploratory. This study aims to fill this research gap by deconstructing and evaluating China’s selenium-enriched policies through the integration of LDA topic modeling and the PMC index model.

### Research design

This study adopted a mixed-methods approach to construct a three-dimensional framework encompassing “policy themes, policy instruments, and policy effectiveness.” Specifically, we employed LDA topic modeling for theme identification, performed content analysis via NVivo software for policy instrument classification, and used the PMC index for assessing internal consistency and structural completeness.

#### Research data and selection criteria.

To ensure the systematic nature and representativeness of the textual analysis of selenium-enriched industry policies, this study adhered to the following fundamental principles during sample construction:

- Principle of Authority: The selected policy texts must be documents formally issued by the Central Committee of the Communist Party of China, the State Council, their constituent departments, as well as people’s governments at provincial and key municipal levels and their functional departments, within their statutory authority and possessing either general binding force or clear guiding effect. The document types primarily include development plans, implementation opinions, action plans, administrative measures, and public notices. Work reports, meeting minutes, leadership speeches, and news reports were excluded from the analysis;
- Principle of Relevance: The core content of the policy texts must be directly and closely related to the development of the “selenium-enriched” industry. Specifically, the texts must contain dedicated chapters, clauses, or explicitly address the “selenium-enriched industry, selenium-enriched products, or selenium-enriched agriculture” as their core subject. The content should encompass one or more key dimensions, such as industrial development planning, resource management, technology R&D, product certification, market promotion, and health orientation. Documents that only tangentially mention the element “selenium” or exhibit weak relevance were excluded to maintain analytical focus.
- Principle of Validity: Only currently valid policies were included in the sample; expired or superseded policies were not considered.

The policy texts for this study were collected by searching authoritative platforms, including the official websites of the Chinese government, the Ministry of Agriculture and Rural Affairs, the Peking University Law Database, the Laws and Regulations Database of China National Knowledge Infrastructure (CNKI), and the official portals of provincial and municipal people’s governments and agricultural departments in key selenium-enriched production areas. The search was conducted using keywords such as “selenium-enriched”, “selenium industry”, “selenium-enriched agricultural products”, “selenium-enriched food”, “selenium resource development” and “naturally selenium-enriched land,” covering the period from 2009 to 2025.

Following an initial compilation of relevant documents, a rigorous three-stage screening process was implemented:

- Preliminary Screening: Duplicate documents and those clearly irrelevant to selenium-enriched industry development were removed.
- Detailed Review: Each document was independently reviewed by two researchers to assess relevance and authority, with disagreements resolved through discussion with a third expert. This stage excluded documents that only superficially mentioned selenium or lacked substantive policy measures.
- Final Verification: The remaining documents were cross-checked against official government sources to confirm validity and issuance dates.

This process ultimately yielded a final sample of 42 selenium-enriched policies for analysis (S1 Table).

#### Research framework.

The three-dimensional analytical framework (Fig 1) systematically evaluates policy structure across three dimensions: policy themes, policy instruments, and policy effectiveness.

**Fig 1:**
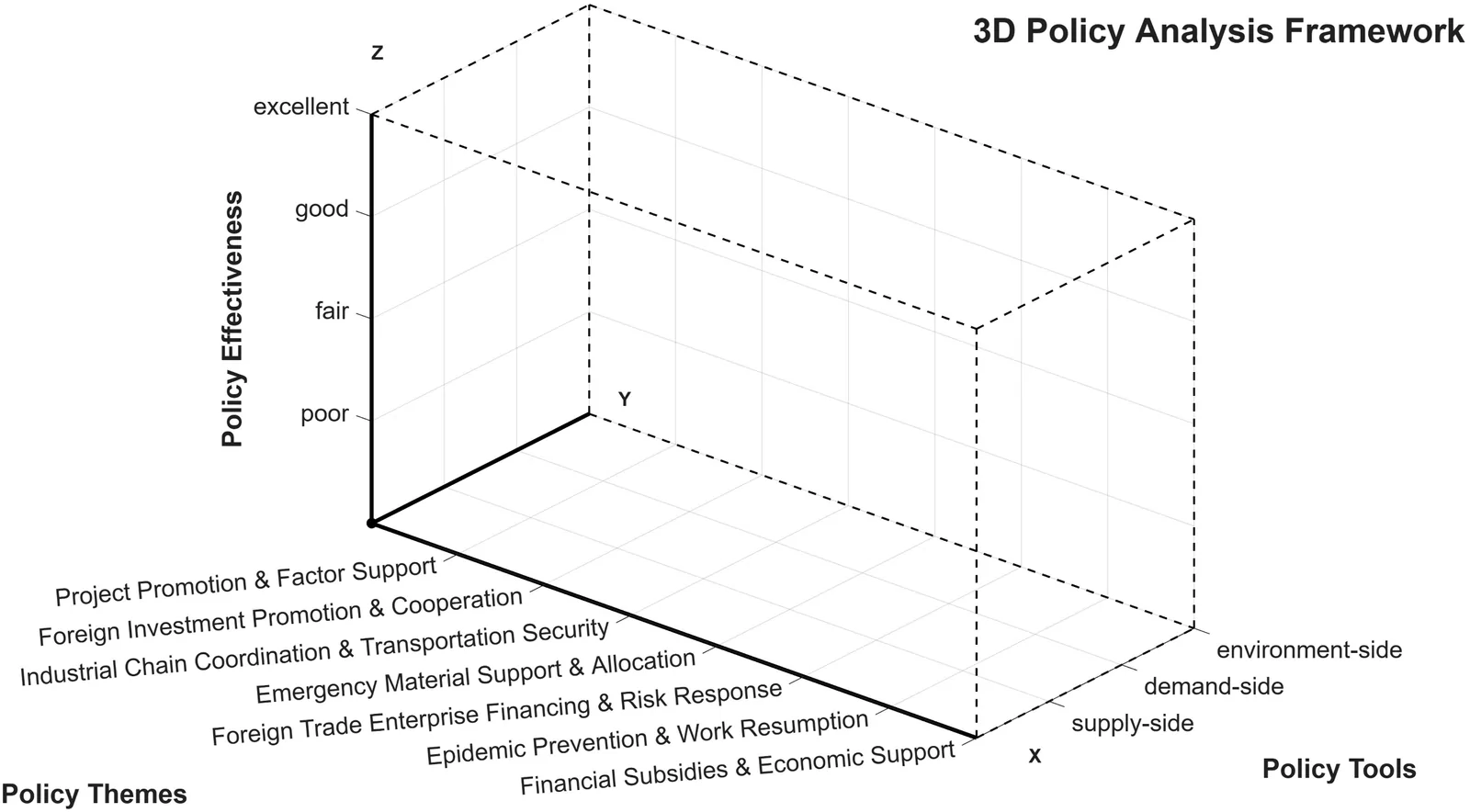
A three-dimensional analytical framework for selenium-enriched policy texts.

The theme dimension (X) captures what the policy system attends to–-the substantive priorities that signal where government chooses to allocate attention and resources. The instrument dimension (Y) captures how government intervenes to act on those priorities–-the supply-, demand-, and environmental-side tools that translate thematic intent into action. The effectiveness dimension (Z) then evaluates how well the resulting design holds together–-the internal consistency and completeness of each policy.

In other words, the framework follows a what → how → how-well progression: themes set the agenda, instruments operationalize it, and the PMC-based effectiveness assessment audits the coherence of the operationalization. Reading the three dimensions together therefore allows us not only to describe each layer but to trace how thematic priorities are (or are not) faithfully and consistently carried through into instrument choices and design quality. This enables a comprehensive assessment of policy characteristics and implementation performance.

- X-axis (Policy Themes): Six categories-cultivation of distinctive selenium-enriched industries and brand support; integrated development of selenium-enriched industries and park construction; industrial chain coordination and talent support for selenium-enriched industries; planning, layout, and financial guarantees for selenium-enriched industries; quality control and technology promotion for selenium-enriched products; and specialized selenium-enriched cultivation and ecological stewardship. The delineation of these six themes is grounded in the quantitative results derived from the LDA topic model applied to the 42 policy texts. The optimal number of topics, K = 6, was determined by calculating topic coherence and validated through manual semantic inspection. Subsequently, the nomenclature for each theme was refined using a modified Delphi method involving eight experts specializing in public policy, agricultural economics, rural governance and industrial policy, nutrition and food hygiene, crop economics, and specialty agriculture regulation. Through two iterative rounds of independent review and anonymous voting, consensus was reached on all six thematic labels. This process ensured that the final nomenclature was both empirically grounded and conceptually coherent, accurately reflecting the core concerns embedded within the policy texts;
- Y-axis (Policy Instruments): Three categories-supply-side, demand-side, and environmental instruments. This classification framework draws on the classic tripartite taxonomy of policy instruments proposed by Rothwell and Zegveld, which has been widely applied in textual analyses across industrial, innovation, and agricultural policies contexts. Its functional orientation aligns with the analytical purpose of this study: to understand how government interventions operate across different stages of the selenium-enriched industrial value chain. To more precisely delineate the specific focal points of policy intervention, this study further refines the three instrument types into 14 sub-instruments (detailed in Table 1), taking into account the dual characteristics of the selenium-enriched industry, which is both health-oriented and agricultural in nature;
- Z-axis (Policy Effectiveness): Four tiers-excellent, good, fair, and bad. This grading standard is based on the evaluation system of the PMC index model developed by Ruiz Estrada. Policies are categorized according to their composite scores across nine primary variables (comprising 37 secondary variables). Specifically, scores of 7–9 are classified as “Excellent”, 5–6.99 as “Good”, 3–4.99 as “Fair”, and 0–2.99 as “Poor”. This tiered classification renders the effectiveness of different policy texts comparable and facilitates the identification of structural deficiencies in policy design. It should be noted that the PMC index measures policy design consistency–-the internal coherence and structural completeness of policy documents–-rather than real-world implementation outcomes.

**Table 1:** Classification and description of policy instruments for the selenium-enriched industry.

Primary Category	Secondary Category	Description	Code
Y1 Supply-side	Selenium resource exploration and foundational data development	Government conducts high-precision soil surveys and multi-dimensional data integration to map the distribution and quality of selenium-enriched resources, providing foundational support for the scientific layout of the industry.	Y1-1
	Construction of selenium-enriched production bases and infrastructure	Government plans and standardizes the development of demonstration bases for specialized cultivation and breeding, agricultural product processing parks, cold chain logistics facilities, and on-site pretreatment centers.	Y1-2
	Specialized fiscal funds and diversified funds investment	Government utilizes fiscal funds, industrial funds, and diversified financial instruments to target capital needs across the industrial chain, lowering the financial barriers to industry development.	Y1-3
	Technology R&D and industry-university-research collaboration	Government promotes research on core selenium-enriched technologies, establishes collaborative platforms, and facilitates the transformation and application of technological achievements.	Y1-4
	Talent cultivation and innovative personnel system support	Government cultivates specialized professionals and optimizes institutional mechanisms to build a skilled workforce, providing talent reserves for industrial development.	Y1-5
	Industry indices and big data services	Government establishes a data monitoring system for the industry and builds smart service platforms to provide data support and efficient services for distribution and decision-making.	Y1-6
Y2 Demand-side	Market expansion and production-marketing linkage services	Government broadens sales channels and creates new consumption scenarios for selenium-enriched products through channel development and scenario innovation.	Y2-1
	Product certification and tiered brand development	Government promotes standard certification and brand system building for selenium-enriched products to enhance market recognition and added value.	Y2-2
	Cultivation of new “Selenium & Wellness Tourism” business models	Government fosters the integration of the selenium-enriched industry with wellness, cultural, and tourism sectors, cultivating specialized consumption scenarios.	Y2-3
	Industrial chain coordination and cultivation of business entities	Government guides the coordinated development of diverse business entities, promotes the extension and integration of industrial chain segments.	Y2-4
Y3 Environmental	Protection of selenium resources and spatial regulation	Government ensures the sustainability of selenium resources through the designation of spatial zones and control of resource utilization, guiding the orderly layout of the industry.	Y3-1
	Whole-process supervision and traceability system construction	Government establishes quality supervision and whole-process traceability mechanisms for selenium-enriched products to regulate market order and ensure product quality.	Y3-2
	Assessment, accountability, and incentive mechanisms	Government combines assessment constraints with policy incentives to promote the implementation of relevant policies.	Y3-3
	Public awareness of selenium and cultural atmosphere cultivation	Government enhances societal awareness and recognition of the selenium-enriched industry through science popularization.	Y3-4

The construction of this three-dimensional framework integrates the objectivity of policy theme identification, the theoretical foundation of policy instrument identification, and the comparability of policy design evaluation, thereby establishing a methodological foundation for the subsequent quantitative analysis.

### Three-dimensional approach (X, Y, Z)

#### X-dimension: LDA topic modeling.

Latent Dirichlet Allocation (LDA) is a generative probabilistic model for discovering latent semantic topics within large collections of text documents [32]. As an unsupervised machine learning technique, LDA assumes that each document is a mixture of multiple topics, and each topic is characterized by a probability distribution over words. This method has been widely adopted in policy text analysis due to its ability to objectively identify hidden thematic structures without prior coding frameworks. To systematically uncover the core areas of concern within selenium-enriched policies, this study employed the LDA topic model for quantitative dimensionality reduction and latent semantic structure identification.

- Text preprocessing

A corpus was constructed from 42 Chinese selenium-enriched agricultural policy documents. All analyses were conducted on the original Chinese texts; no machine translation or English-language substitutes were used at any stage of the text mining process. First, the Jieba tokenizer in Python was applied to segment the full texts into individual word units. To ensure accurate segmentation of domain-specific terminologies, a custom dictionary was constructed for policy-relevant vocabulary (e.g., “selenium-enriched”, “functional agriculture”, “selenium resources”), which was incorporated into the tokenization process to enhance segmentation accuracy. Subsequently, a standard Chinese stopword list was applied to remove semantically empty function words, conjunctions, and punctuation marks. This list was further refined to exclude high-frequency but semantically uninformative administrative terms such as those denoting generic actions or procedural expressions. After completing the cleaning process described above, the documents were converted into structured text corpora, preparing them for subsequent topic modeling.

- Determining the optimal number of topics

LDA topic modeling was implemented using the Gensim library in Python. The hyperparameters were configured as follows: the document-topic prior α was set to ‘auto’, allowing it to adaptively learn the optimal prior distribution from the corpus during training; the topic-word prior β was left at its default symmetric prior of 1/K to maintain a balanced distribution of topic words; the number of training passes was set to 10 to ensure sufficient model convergence; and a fixed random seed was used to guarantee reproducibility. Under this parameter framework, we proceeded to determine the optimal number of topics K.

Determining the optimal number of topics (the K value) is a critical decision in LDA modeling. A low value of K may result in topics that are overly broad and semantically ambiguous, while a high value can lead to topic fragmentation, diminishing their value for macro-level synthesis. To address this issue, this study employed coherence as a quantitative evaluation metric. A higher topic coherence score indicates that the words within a topic are highly semantically related, that topics are well-distinguished from one another, and that the topics possess greater explanatory power. This study calculated the coherence scores for all models with topic numbers K ranging from 1 to 10 and plotted the coherence-topic number curve, as shown in Fig 2, and the topic coherence scores for models with K ranging from 1 to 10 are presented in S2 Table.

**Fig 2:**
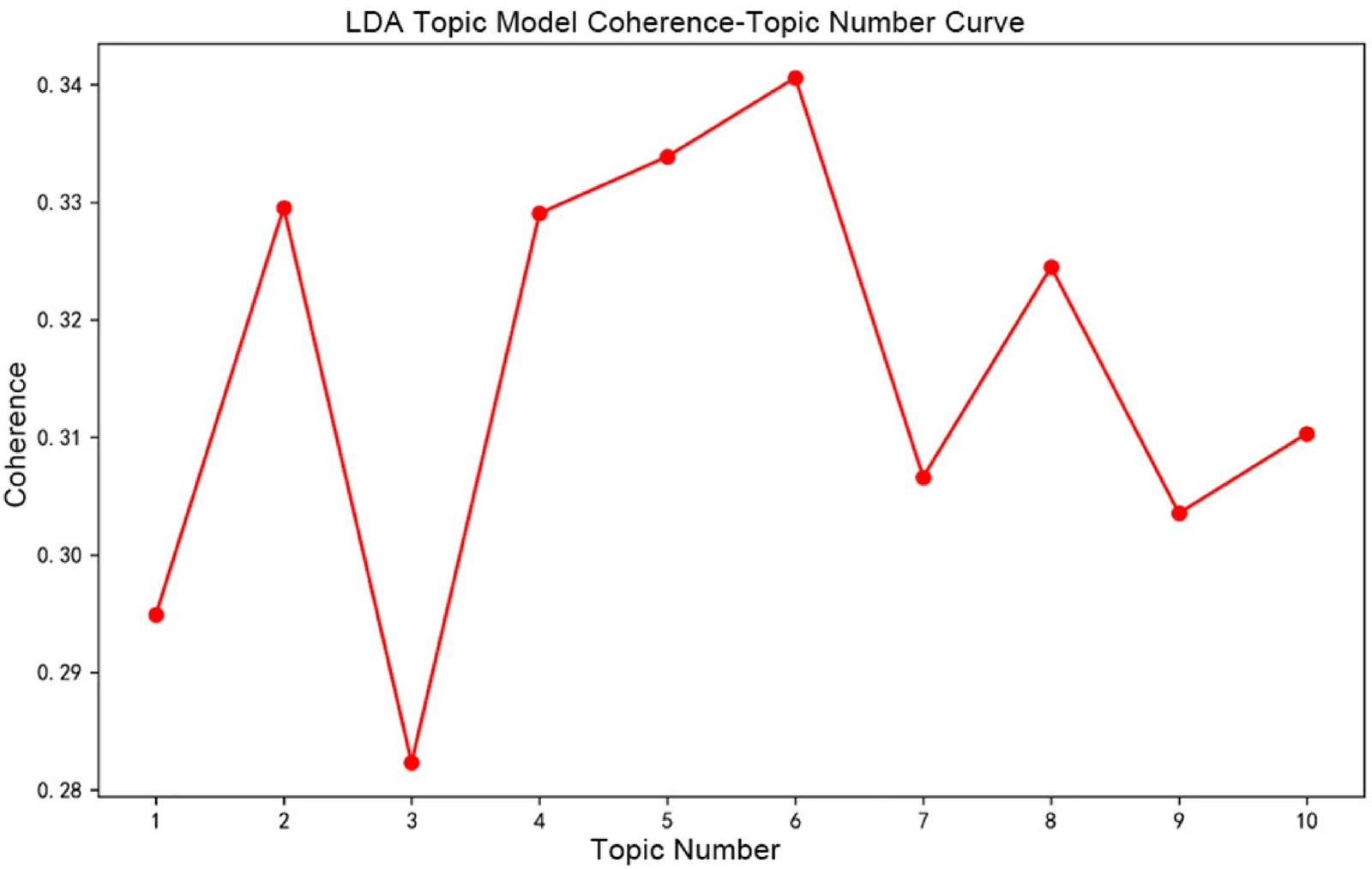
Coherence curve of the LDA topic model.

As shown in Fig 2, the coherence score reached a notable peak at K = 6. To validate this quantitative result, we manually inspected the top-frequency words for each topic, examining the logical relatedness of core words within each policy context and verifying whether semantic boundaries between topics were clearly distinguishable. The manual validation confirmed that K = 6 best balances intra-topic cohesion and inter-topic discrimination, with each topic exhibiting clear policy implications and interpretable labels. Accordingly, K = 6 was selected as the optimal number of topics, under which the LDA model most effectively extracts and summarizes the core concerns of the selenium-enriched policy texts from 2009 to 2025.

#### Y-dimension: Content analysis method.

Content analysis is a widely employed method in policy studies for systematically categorizing and quantifying textual information. Its core premise is that the frequency and pattern of specific content elements can reveal underlying priorities and strategic orientations of policy designers. In this study, we applied content analysis to identify and classify the policy instruments embedded in the 42 selenium-enriched policy documents, using NVivo 11.0 Plus software to support the coding process.

The classification framework was grounded in the functional typology proposed by Rothwell and Zegveld, which distinguishes policy instruments into three categories–-supply-side, demand-side, and environmental–-according to their respective roles in shaping industrial development. This framework was selected over alternative classifications for two reasons. First, its functional orientation aligns with the analytical purpose of this study: to understand how government interventions operate across different stages of the selenium-enriched industrial value chain. Second, the tripartite structure has been extensively validated in agricultural and industrial policy contexts, making it directly applicable to the selenium-enriched sector, which spans both agricultural production and health-oriented consumption.

The three categories differ fundamentally in their mechanisms of influence. Supply-side instruments focus on augmenting the availability of production factors–-financial resources, physical infrastructure, technology, talent, and information services. These instruments directly lower the barriers to entry and expansion for firms and producers in the selenium-enriched industry. Demand-side instruments, in contrast, aim to shape and expand market conditions by promoting product certification, brand recognition, market access, and new consumption scenarios such as wellness tourism. Their logic is to generate market signals that pull industrial activities toward higher value-added segments. Environmental instruments operate at a systemic level, establishing the regulatory, institutional, and cultural context within which the selenium-enriched industry operates.

This study adopted the tripartite taxonomy of supply-side, environmental, and demand-side policy instruments. Tailoring this framework to the characteristics of selenium-enriched policies, it further refined these categories into 14 sub-instruments. The specific policy instruments and their corresponding explanations are presented in Table 1, establishing the coding basis for subsequent quantitative analysis.

Based on the coding scheme described above, we systematically coded the 42 policy documents following a two-stage procedure. In the first stage, each policy document was read line-by-line and relevant text segments were assigned to the appropriate sub-instrument categories. In the second stage, conducted two weeks later, the researcher systematically reviewed all assignments against the coding manual to ensure consistency and correct any ambiguous classifications. Cases that remained uncertain were discussed with a second researcher to reach a consensus.

#### Z-dimension: Policy modeling consistency (PMC) index model.

The policy effectiveness dimension aims to evaluate the design quality of individual policy texts, encompassing the clarity of their objectives, the coherence of their measures, the comprehensiveness of their perspectives, and the completeness of their strategic functions.

The PMC index model was originally developed by Ruiz Estrada, grounded in the core methodological premise of “Omnia Mobilis”–-the principle that policy evaluation should adopt an inclusive analytical lens, incorporating as many relevant factors as possible rather than restricting assessment to a narrow set of dimensions [29]. As a text-driven evaluation tool, the PMC model systematically assesses the coverage and internal coherence of policy documents across multiple dimensions through a hierarchical indicator system. Compared with alternative evaluation approaches such as the entropy weight method or the analytic hierarchy process (AHP), the PMC model employs standardized binary scoring combined with equal weighting aggregation, which effectively minimizes subjective bias in the evaluation process.

In determining the PMC evaluation variables, this study adhered to three guiding principles: comprehensiveness, relevance, and structural clarity. Comprehensiveness requires that the indicators cover the major dimensions of policy design; relevance ensures alignment with the specific characteristics of the selenium-enriched industry; and structural clarity demands a logically coherent, non-overlapping indicator hierarchy. The selection of specific variables drew upon empirically validated indicator systems documented in the existing PMC literature and was refined through multiple rounds of discussion and cross-validation by the research team. This process ultimately yielded a PMC evaluation framework comprising 9 primary indicators and 37 secondary indicators for selenium-enriched policies, as presented in Table 2.

**Table 2:** PMC index system for selenium-enriched policy evaluation.

Primary variable	Secondary variable	Basis for assignment	Source
X1 Policy nature	X1:1 prediction	Whether the policy contains predictive content; 1 for yes, 0 for no	[33]
	X1:2 suggestion	Whether the policy offers suggestions; 1 for yes, 0 for no	
	X1:3 regulation	Whether the policy has regulatory functions; 1 for yes, 0 for no	
	X1:4 guidance	Whether the policy provides guidance; 1 for yes, 0 for no	
	X1:5 support	Whether the policy includes supportive measures; 1 for yes, 0 for no	
	X1:6 diagnosis	Whether the policy contains diagnostic content; 1 for yes, 0 for no	
X2 Policy timeliness	X2:1 short-term	Whether the policy addresses 1–3-year objectives; 1 for yes, 0 for no	[29]
	X2:2 medium-term	Whether the policy addresses 3–5-year objectives; 1 for yes, 0 for no	
	X2:3 long-term	Whether the policy addresses objectives beyond 5 years; 1 for yes, 0 for no	
X3 Policy content	X3:1 resources and planning	Coverage of selenium resource surveys, industrial planning, spatial layout; 1 for yes, 0 for no	Based on policy text analysis
	X3:2 production and bases	Coverage of selenium-enriched agricultural production, standardized bases, park construction; 1 for yes, 0 for no	
	X3:3 technology and standards	Inclusion of R&D, standard setting, testing and certification, quality traceability; 1 for yes, 0 for no	
	X3:4 industry and integration	Promotion of industrial chain extension, industrial integration, new business models; 1 for yes, 0 for no	
	X3:5 brand and market	Deployment of brand building, promotion, marketing, channel expansion; 1 for yes, 0 for no	
X4 Policy objectives	X4:1 industrial scale	Clear targets for output value, base area, etc.; 1 for yes, 0 for no	Based on policy text analysis
	X4:2 quality and brand	Clear targets for product quality, number of certifications, etc.; 1 for yes, 0 for no	
	X4:3 social benefits	Mention of farmer income increase, employment generation, health promotion; 1 for yes, 0 for no	
	X4:4 ecological benefits	Emphasis on resource protection, green development, sustainable utilization; 1 for yes, 0 for no	
X5 Policy recipients	X5:1 production and business entities	Coverage of farmers, cooperatives, agricultural enterprises; 1 for yes, 0 for no	[34]
	X5:2 research institutions	Targeting universities, research institutes, R&D platforms; 1 for yes, 0 for no	
	X5:3 government departments	Specification of responsibilities for governments and departments at various levels; 1 for yes, 0 for no	
	X5:4 consumers and the public	Involvement of consumer education, science popularization, public participation; 1 for yes, 0 for no	
X6 Mode of action	X6:1 mandatory	Presence of mandatory measures; 1 for yes, 0 for no	[35]
	X6:2 service-oriented	Presence of service-oriented measures; 1 for yes, 0 for no	
	X6:3 incentive-based	Presence of incentive-based measures; 1 for yes, 0 for no	
X7 Support mechanisms	X7:1 financial support	Involvement of fiscal investment, financial support, special funds; 1 for yes, 0 for no	Based on policy text analysis
	X7:2 organizational support	Establishment of leading groups, clarification of responsibility mechanisms; 1 for yes, 0 for no	
	X7:3 talent and technology support	Involvement of talent introduction, training, R&D platform construction; 1 for yes, 0 for no	
	X7:4 supervision and assessment	Establishment of supervision, assessment, evaluation, accountability mechanisms; 1 for yes, 0 for no	
X8 Policy evaluation	X8:1 adequate basis	Whether the policy has a sufficient basis; 1 for yes, 0 for no	[29]
	X8:2 clear objectives	Whether the policy has clear objectives; 1 for yes, 0 for no	
	X8:3 scientifically sound design	Whether the policy design is scientifically sound; 1 for yes, 0 for no	
	X8:4 detailed content	Whether the policy content is detailed; 1 for yes, 0 for no	
X9 Issuing level authority	X9:1 national level	Issued by national authorities; assigned value 1	[36]
	X9:2 provincial level	Issued by provincial authorities; assigned value 0.8	
	X9:3 municipal level	Issued by municipal authorities; assigned value 0.6	
	X9:4 county level	Issued by county authorities; assigned value 0.4	

The scoring followed the standard PMC procedure: each secondary variable was assigned a value of 1 if the corresponding policy element was present, and 0 otherwise. Primary variable scores were calculated as the arithmetic mean of their constituent secondary variables, and the final PMC index was obtained by summing all nine primary variable scores. Specifically, for the issuing authority (X9), four mutually exclusive secondary indicators are delineated according to administrative levels-national, provincial, municipal, and county. To ensure that comparative evaluation can be conducted, the parameters for these four indicators are set within the range of [0,1] and assigned values in descending order. Beyond this, all other secondary indicators adhere to a [0,1] distribution.

The calculation of the PMC index follows a four-step procedure.

1. Through textual analysis, binary values (0/1) are assigned to secondary variables, as shown in Equations (1) and (2).
2. Scores for primary variables are calculated as the average of their corresponding secondary variables, following Equation (3).
3. Equation (4) is applied to sum the scores of all primary variables, thereby deriving the PMC index for each policy.
4. Policies are then classified according to the grading standards presented in Table 3.

$$\begin{array}{c} X\sim N(0,1) \end{array}$$ (1)

$$\begin{array}{c} X=\{XR:[0,1]\} \end{array}$$ (2)

$$\begin{array}{c} PI_{t}=\left\{ \begin{array}{cc} X_{t}\left(\sum_{j=1}^{n}\frac{X_{tj}}{T(X_{tj})}\right), & t=1,2,...,8\\ X_{9}, & t=9 \end{array}\right. \end{array}$$ (3)

$$\begin{array}{cc} PMC & =X_{1}\left(\sum_{j=1}^{6}\frac{X_{1j}}{6}\right)+X_{2}\left(\sum_{j=1}^{3}\frac{X_{2j}}{3}\right)+X_{3}\left(\sum_{j=1}^{5}\frac{X_{3j}}{5}\right)\\ & +X_{4}\left(\sum_{j=1}^{4}\frac{X_{4j}}{4}\right)+X_{5}\left(\sum_{j=1}^{4}\frac{X_{5j}}{4}\right)+X_{6}\left(\sum_{j=1}^{3}\frac{X_{6j}}{3}\right)\\ & +X_{7}\left(\sum_{j=1}^{4}\frac{X_{7j}}{4}\right)+X_{8}\left(\sum_{j=1}^{4}\frac{X_{8j}}{4}\right)+PI_{9} \end{array}$$ (4)

$$\begin{array}{c} PMC=[\begin{array}{ccc} PI_{1} & PI_{2} & PI_{3}\\ PI_{4} & PI_{5} & PI_{6}\\ PI_{7} & PI_{8} & PI_{9} \end{array}] \end{array}$$ (5)

**Table 3:** Grading standards for policy evaluation.

PMC index values	0-2.99	3-4.99	5-6.99	7-9
**Evaluation levels**	Bad	Fair	Good	Excellent

To further visualize the performance disparities across dimensions, this study employed PMC surface plots to present the evaluation results as shown in Equation (5). These plots display the score profiles of individual policies across the nine primary indicators in a three-dimensional matrix format, where concave areas on the surface indicate potential weaknesses in policy design, thereby facilitating targeted optimization recommendations in subsequent discussions.

## Results and discussion

### X-dimensional analysis: Policy themes

To systematically uncover the core areas of concern within selenium-enriched policies, this study employed the LDA topic model for preliminary quantitative dimensionality reduction, identifying latent semantic structures embedded in the policy texts. Building on this, we further applied the Gioia methodology to conduct rigorous qualitative induction and theoretical refinement of the topics and characteristic terms generated by the model. This ensures that the thematic classification is both grounded in the original data and possesses conceptual clarity and theoretical insight [37]. Specifically, the analysis follows the “first-order concepts-second-order themes-aggregate dimensions” logic proposed by Gioia et al.: First, the characteristic terms with the highest weights under each topic (as shown in Table 4), along with their typical policy clauses, are taken as first-order concepts, preserving the linguistic characteristics of the original data. Second, through continuous comparison and iterative induction, first-order concepts with similar semantics and practical orientations are aggregated to form second-order themes. Finally, further abstraction at the theoretical level aggregates interrelated second-order themes into six core policy themes (i.e., the six categories presented in Table 4).

**Table 4:** Classification of policy themes in selenium-enriched policies.

No.	Theme	Keywords
1	Cultivation of distinctive selenium-enriched industries and brand support	management, development, quality, tea, livestock, support, investment, promotion, trademark, marketing
2	Integration of selenium-enriched industries and park development	food, culture, innovation, tourism, technology, development, industrial chain, health, cluster, industrial park
3	Industrial chain coordination and talent support for selenium-enriched industries	industrial chain, role, innovation, coordination, organization, management, talent, quality, fully leverage, application
4	Planning and financial guarantees for selenium-enriched industries	development, funding, planning, investment, prevention and control, food, materials, health, coordination, tourism
5	Quality control and technology promotion for selenium-enriched products	function, testing, cultivation, cooperation, innovation, center, edible fungi, management, sales, certification
6	Specialized selenium-enriched cultivation and ecological base construction	quality, walnut, breeding, project, konjac, pig, improved variety, public, ecology, propagation

LDA topic modeling was applied to the 42 policy texts. The optimal number of topics (K = 6) was determined through text preprocessing and coherence curve analysis (Fig 3). Table 4 presents the six themes and their characteristic terms.

**Fig 3:**
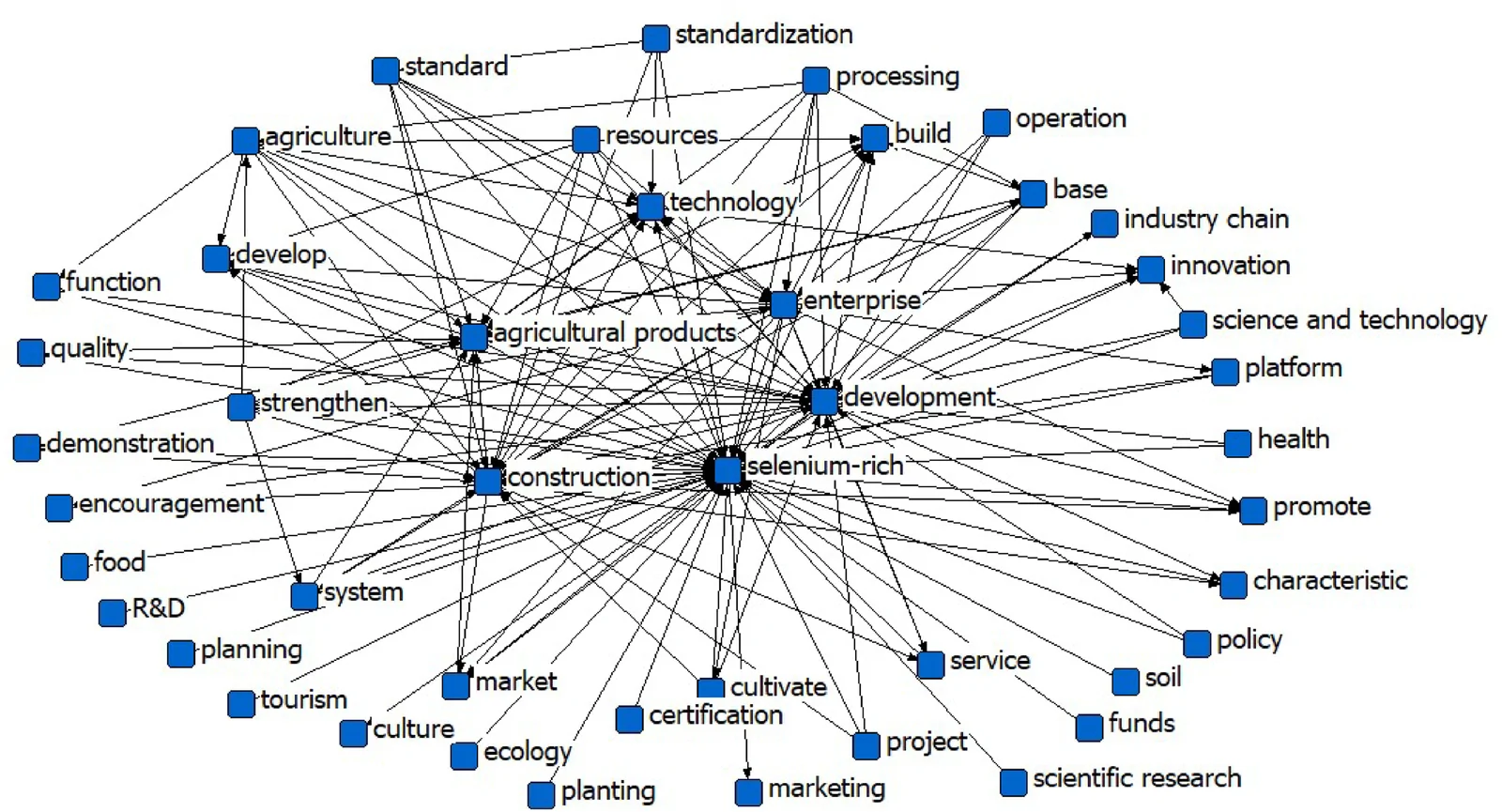
Semantic network map of thematic keywords in selenium-enriched policies.

Theme 1 (Cultivation of distinctive selenium-enriched industries and brand support) and Theme 2 (Integration of selenium-enriched industries and park development) function as dual-core drivers, reflecting a progressive logic that emphasizes both “consolidating product competitiveness” and “expanding industrial added value”. Theme 3 (Industrial chain coordination and talent support for selenium-enriched industries), Theme 4 (Planning and financial guarantees for selenium-enriched industries), and Theme 5 (Quality control and technology promotion for selenium-enriched products) collectively establish a comprehensive support network integrating talent, capital, technology, and quality. Theme 6 (Specialized selenium-enriched cultivation and ecological base construction) focuses on the production source and ecological foundation, indicating policy attention to specialized cultivation and sustainable production practices.

To further validate the rationale of the identified themes and intuitively present the semantic relationships and discourse structures among thematic terms, this study employed ROSTCM6 software to conduct a social network and semantic network analysis of the selenium-enriched policy texts. The semantic network of thematic terms derived from these policies is presented in Fig 3.

Fig 3 reveals that “selenium” forms a highly dense cooccurrence network with terms such as “development”, “construction”, “base”, and “agricultural products”, corresponding to the industrial cultivation main thread embedded in Theme 1 (Cultivation of distinctive selenium-enriched industries and brand support). Terms including “standard”, “quality”, “technology” and “certification” are closely connected to the core nodes, reflecting the brand dimension of Theme 1 and the quality control and technological support connotations of Theme 5 (Quality control and technology promotion for selenium-enriched products). Words such as “tourism” and “health” form a secondary cluster, corresponding to the integration orientation of Theme 2 (Integration of selenium-enriched industries and park development). Terms including “funds”, “scientific research”, “platform”, “industrial chain” and “planning” constitute another cluster, aligning with the factor guarantees of Theme 3 (Industrial chain coordination and talent support for selenium-enriched industries) and Theme 4 (Planning and financial guarantees for selenium-enriched industries). Additionally, terms such as “base”, “cultivation”, “breeding”, “ecology” and “soil” exhibit connections to the core nodes, underscoring the specialized cultivation and base construction orientation of Theme 6 (Specialized selenium-enriched cultivation and ecological base construction).

The network structure validates the rationality of the six identified themes and intuitively illustrates the fundamental configuration of the policy discourse: originating from selenium resources as the foundational point, progressing toward industrial cultivation, quality control, and brand building, and further extending to industrial integration, factor guarantees, and ecological stewardship.

With the optimal number of topics determined (K = 6), the LDA model was trained on the preprocessed corpus using this parameter. The results of the LDA model were subsequently visualized using the “pyLDAvis” library, which intuitively illustrates the relative distances and distribution among the topics, as shown in Fig 4, and the detailed pyLDAvis visualization for each of the six topics, along with the corresponding top 30 characteristic terms, is provided in S1 Fig.

**Fig 4:**
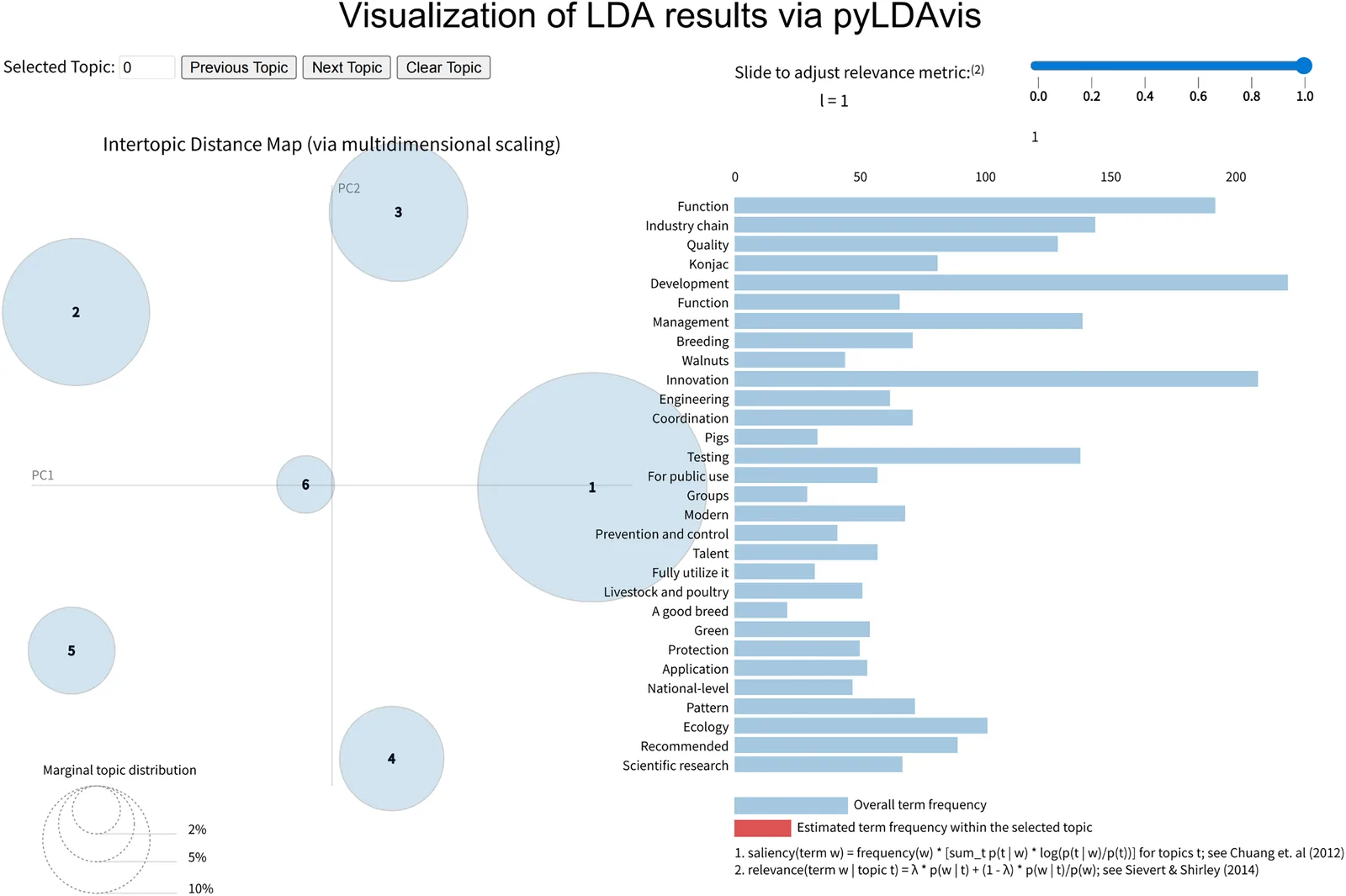
Visualization of LDA results via pyLDAvis.

The bubble area intuitively reflects the relative weight and frequency of each theme within the overall policy corpus. A larger bubble area indicates more concentrated policy attention and resource allocation toward a given theme, signifying its more central position within the current policy system.

From the distribution of themes, Theme 1 (Cultivation of distinctive selenium-enriched industries and brand support) occupies the absolute core with the most prominent bubble area. This indicates that the primary focus of the policy system is on transforming selenium resource advantages into market-competitive products and brands. Themes 2 (Integration of selenium-enriched industries and park development) and 3 (Industrial chain coordination and talent support for selenium-enriched industries) constitute key secondary clusters supporting the core objective. Theme 2 emphasizes extending the industrial chain and enhancing added value through the “Selenium+” model (e.g., health, cultural tourism), while Theme 3 focuses on organizational optimization and human capital development within the industry. Together, they point toward industrial upgrading and systematic development, reflecting an important policy orientation aimed at transitioning the industry from “existence” to “excellence.”

However, the model results also reveal a significant imbalance in the distribution of policy themes. Themes 4 (Planning and financial guarantees for selenium-enriched industries), 5 (Quality control and technology promotion for selenium-enriched products), and 6 (Specialized selenium-enriched cultivation and ecological base construction) exhibit relatively lower policy visibility. This reflects that areas intended to provide long-term, foundational support for the industry have not yet achieved a level of prominence and resource allocation intensity within the current policy agenda commensurate with short-term objectives like industrial and market cultivation.

This thematic structure clearly delineates the phased characteristic of the current policy system: it is stronger in industrial initiation and market breakthroughs, yet weaker in systemic construction and long-term foundational development. In the early stages of industrial development, focusing on brand building and market expansion is both practically reasonable and necessary. However, if this thematic configuration persists over the long term, it may imply that policies have yet to formulate sufficiently forward-looking and robust strategic responses to the deeper foundations of sustainable industrial development.

### Y-dimensional analysis: Policy instruments

By integrating the selenium-enriched policy texts and employing Nvivo 11.0 Plus software for coding analysis, we were able to systematically examine the application of the three types of policy instruments-supply-side, demand-side, and environmental-within selenium-enriched policies. The analysis yielded a total of 3616 coded references, with the frequency and percentage distribution summarized in Table 5. Based on the coding and frequency statistics of selenium-enriched industry policy texts, it is evident that the current policy system comprehensively employs supply-side, demand-side, and environmental policy instruments. However, notable disparities exist in their internal structure and focal points of utilization. In terms of overall distribution, demand-side policy instruments account for the highest proportion (37.97%), followed by supply-side instruments (36.48%), while environmental instruments (25.55%) serve as essential regulatory and supporting complements, as illustrated in Fig 5.

**Table 5:** Frequency statistics of policy instrument usage in selenium-enriched policies.

Policy instrument category	Specific policy instrument	Frequency by subcategory	Share by subcategory (%)	Share by main category (%)
Y1 Supply-side	Selenium resource exploration and foundational data development	102	2.82	36.48
	Construction of selenium-enriched production bases and infrastructure	418	11.56	
	Specialized fiscal funds and diversified investment and financing support	206	5.70	
	Technology R&D and the establishment of industry-university-research collaboration platforms	387	10.70	
	Talent cultivation and innovative personnel system support	108	2.99	
	Selenium-enriched industry indices and big-data services	98	2.71	
Y2 Demand-side	Market expansion and production-marketing linkage services for selenium-enriched products	363	10.04	37.97
	Product certification and tiered brand development	353	9.76	
	Cultivation of new “Selenium & Wellness Tourism” business models	131	3.62	
	Industrial chain coordination and cultivation of business entities	526	14.55	
Y3 Environmental	Protection of selenium resources and spatial regulation	136	3.76	25.55
	Whole-process supervision and traceability system construction for selenium-enriched products	312	8.63	
	Assessment, accountability, and incentive mechanisms for the selenium-enriched industry	216	5.97	
	Public awareness of selenium and cultivation of a supportive cultural atmosphere	260	7.19	
**Total**	–	3616	100	100

**Fig 5:**
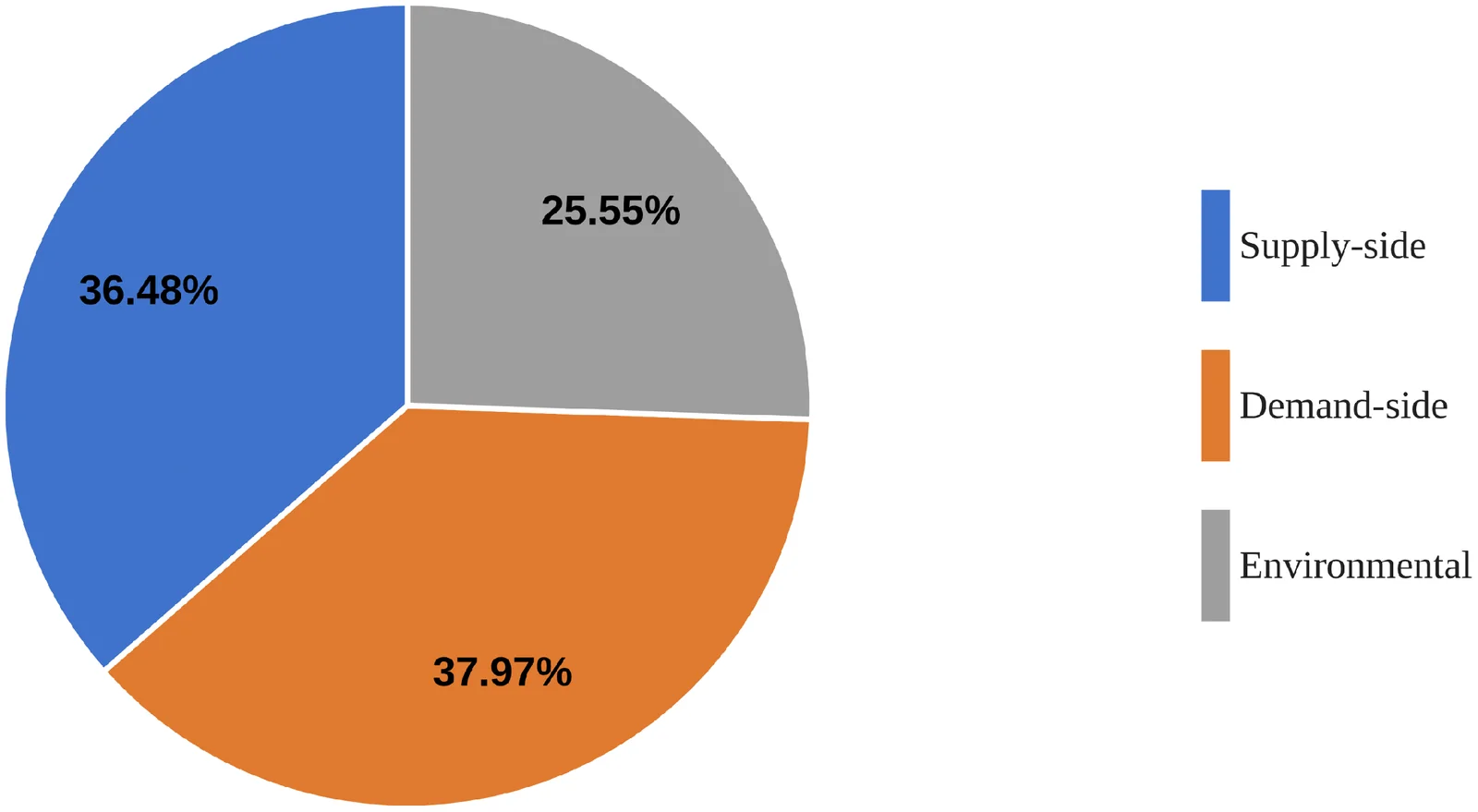
Distribution of policy instrument types.

The data reveal that the current policy system for the selenium-enriched industry exhibits an overall pattern characterized by “equal emphasis on supply-side and demand-side instruments, yet with relatively limited support from environmental instruments.” As illustrated in Fig 5, demand-side policy instruments account for the highest proportion (37.97%), followed closely by supply-side instruments (36.48%), with these two categories jointly constituting the dual mainstay of policy intervention. Environmental policy instruments (25.55%) primarily fulfill regulatory and safeguarding functions.

Fig 6 presents the detailed distribution of all 14 sub-instruments across the three categories, with sub-instruments grouped by main category for visual comparison.

**Fig 6:**
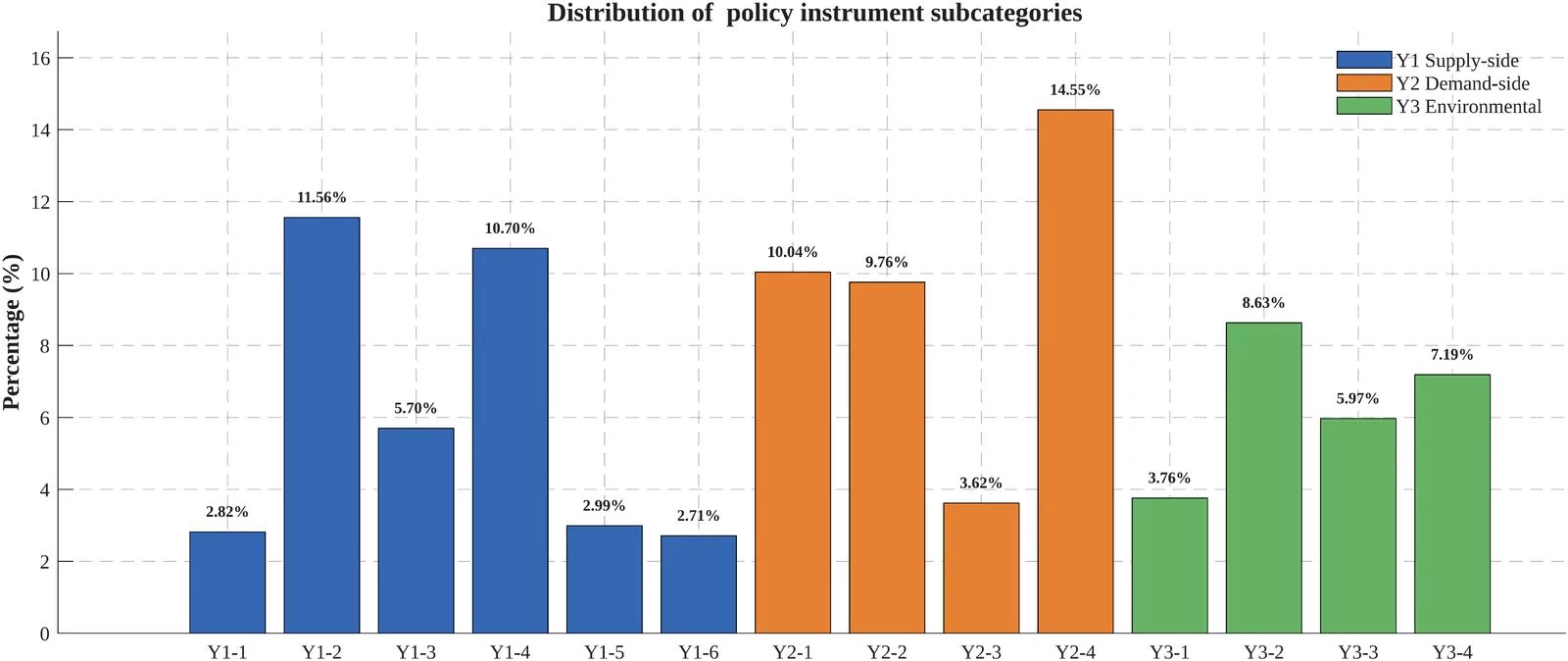
Distribution of policy instrument subcategories.

Within the supply-side category (36.48%, 1319 coded references), Construction of selenium-enriched production bases and infrastructure (11.56% of total) and Technology R&D and the establishment of industry-university-research collaboration platforms (10.70% of total) constitute the dominant sub-instruments, underscoring the policy system’s strong emphasis on physical infrastructure and technological capacity. By contrast, non-physical supports–-including Talent cultivation and innovative personnel system support (2.99%), Selenium resource exploration and foundational data development (2.82%), and Selenium-enriched industry indices and big-data services (2.71%)–-remain notably underutilized, indicating a strategic deficiency in building intangible assets essential for long-term industrial competitiveness.

Within the demand-side category (37.97%, 1373 coded references), Industrial chain coordination and cultivation of business entities (14.55% of total) dominates, reflecting a strategy that strengthens market supply capacity from the production side. Market expansion and production-marketing linkage services for selenium-enriched products (10.04%) and Product certification and tiered brand development (9.76%) jointly constitute a complementary policy package for market value realization and trust building. Cultivation of new “Selenium & Wellness Tourism” business models (3.62%) remains the least utilized, indicating that direct consumer engagement and health-oriented consumption scenarios are still in early developmental stages.

Within the environmental category (25.55%, 924 coded references), Whole-process supervision and traceability system construction for selenium-enriched products (8.63% of total) represents the most frequently employed instrument, followed by Public awareness of selenium and cultivation of a supportive cultural atmosphere (7.19%) and Assessment, accountability, and incentive mechanisms for the selenium-enriched industry (5.97%). Notably, Protection of selenium resources and spatial regulation accounts for only 3.76% of the total, revealing a structural characteristic of stronger emphasis on ex-post supervision and in-process control relative to ex-ante source protection and forward-looking spatial planning.

In summary, the instrument configuration exhibits several structural features: (1) dual dominance of supply- and demand-side instruments (74.45% combined), with environmental instruments playing a complementary regulatory role; (2) within supply-side, a strong hardware orientation with relative neglect of intangible factor inputs; (3) within demand-side, a production-side logic that emphasizes business entity cultivation over direct consumer engagement; and (4) within environmental instruments, a reactive orientation that prioritizes supervision and accountability over proactive resource conservation.

### Z-dimensional analysis: Policy effectiveness

To balance analytical depth with feasibility, 10 representative policies were selected from the 42 collected policy documents using a stratified approach. The selection prioritized coverage across three dimensions: issuing authority level, policy type, and issuance year, ensuring that the subset reflected the diversity of the broader corpus. Based on the PMC index model, the PMC index value of each policy text is calculated, reflecting the degree of deviation between the evaluated policy and an ideal policy. A higher PMC index indicates that the policy has more comprehensive coverage and better internal coherence across the various dimensions of the evaluation system. Conversely, a lower PMC index suggests a relatively lower policy grade. The calculated PMC index and concavity index for the 10 representative selenium-enriched policies are summarized in Table 6. The detailed binary scoring sheets for all 10 representative policies across the 33 secondary variables (X1-X8) and the tiered weights for X9 are provided in S3 Table (X1-X4) and S4 Table (X5-X9).

**Table 6:** Summary of PMC index scores for 10 selenium-enriched policies.

First-level variable	P3	P6	P11	P17	P22	P23	P26	P35	P40	P42	Mean value
X1	0.5	0.67	0.67	1	0.67	0.5	0.5	0.67	0.5	0.83	0.65
X2	0.33	0.33	0.33	0.33	0.33	0.33	0.67	0.67	0.33	0.33	0.4
X3	1	1	1	1	0.4	0.8	1	1	0.8	1	0.9
X4	0.75	1	0.75	1	0.75	0.75	1	1	0.75	0.5	0.83
X5	0.75	1	1	0.75	0.75	1	1	1	1	0.75	0.9
X6	0.67	1	1	1	0.67	0.67	0.67	1	0.67	1	0.84
X7	0.5	1	1	0.5	0.25	0.75	1	1	0.75	1	0.78
X8	1	1	1	1	1	1	1	1	1	0.75	0.98
X9	0.8	0.6	0.8	0.4	0.6	0.8	0.6	0.8	0.4	0.6	0.64
PMC index	6.3	7.6	7.55	6.98	5.42	6.6	7.44	8.14	6.2	6.76	6.9
Concavity index	2.7	1.4	1.45	2.02	3.58	2.4	1.56	0.86	2.8	2.24	2.1
Policy grade	Good	Excellent	Excellent	Good	Good	Good	Excellent	Excellent	Good	Good	–

As shown in the summary of PMC index scores for the 10 selenium-enriched policies presented in Table 6, four policy texts (P6, P11, P26, P35) are rated as excellent, while six (P3, P17, P22, P23, P40, P42) are rated as good. The average PMC index across the 10 policies is 6.9, falling within the “Good” consistency range. This indicates that the overall design of China’s selenium-enriched policy documents is relatively coherent and well-structured, though there remains considerable room for improvement in specific dimensions.

It is important to emphasize that the PMC scores reported in this section reflect the level of internal consistency and structural completeness of policy documents–-that is, the degree to which a policy text comprehensively addresses the evaluation criteria–-rather than a judgment on the overall quality or effectiveness of any single policy paper. A higher PMC score indicates that a policy is more comprehensively designed across the evaluated dimensions, but does not necessarily imply superior implementation outcomes or real-world effectiveness.

Fig 7 presents the PMC surface plot for the mean scores of all nine primary variables, providing a visual overview of the overall policy performance across dimensions. The surface plot reveals the average design profile of selenium-enriched policies, with concave areas indicating dimensions where the policy system, on average, exhibits weaker performance.

**Fig 7:**
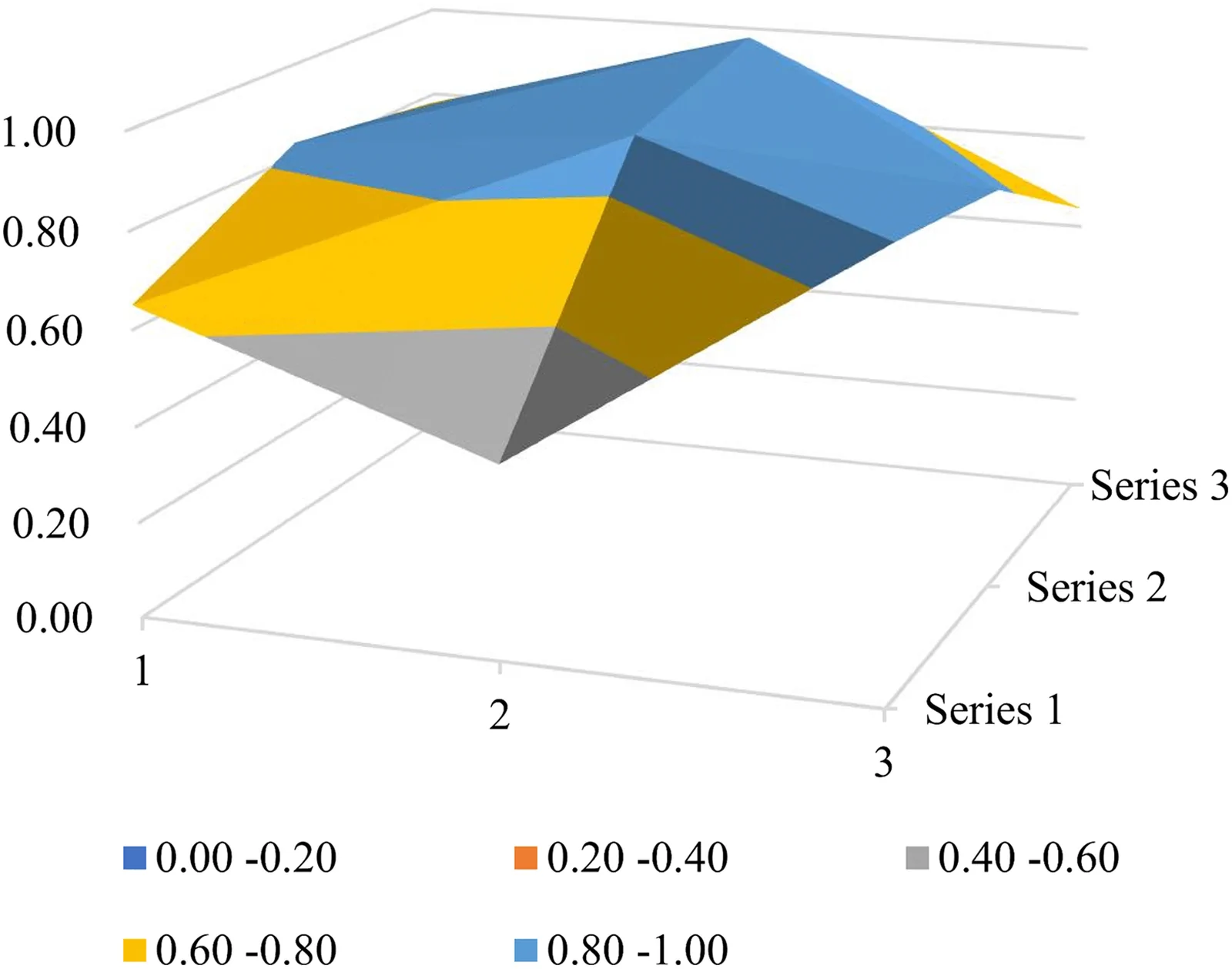
PMC surface plot for mean scores.

To contextualize the mean-surface results, the following patterns merit attention. According to Table 6, the mean scores of the nine primary variables, in descending order, were: *X*8 (0.98) > *X*3 (0.9) = *X*5 (0.9) > *X*6 (0.84) > *X*4 (0.83) > *X*7 (0.78) > *X*1 (0.65) > *X*9 (0.64) > *X*2 (0.4).This ranking reveals a clear bifurcation: three dimensions consistently outperform the others, while two dimensions consistently underperform relative to the sample average.

(1) Highest-scoring dimensions. The highest mean scores were observed for X8 Policy evaluation (0.98), X3 Policy content (0.9), and X5 Policy recipients (0.9). X8 reflects that all policies achieved strong textual standardization–-all but one scored 1 on this indicator–-indicating that selenium-enriched policies are generally well-formulated in terms of clarity, detail, and normative quality. X3 and X5 suggest that policies comprehensively address industrial elements–-covering production, processing, branding, and market cultivation–-and clearly identify diverse stakeholders, including farmers, cooperatives, enterprises, research institutions, and government departments.
(2) Mid-range dimensions. X6 Mode of action (0.84) and X4 Policy objectives (0.83) performed relatively well, suggesting that selenium-enriched policies generally deploy a mix of mandatory, service-oriented, and incentive-based measures with clearly defined developmental targets. X7 Support mechanisms (0.78) achieved a moderate mean score, yet notable variation exists across individual policies. Policies P6, P11, P26, P35, and P42 achieved full scores by comprehensively addressing financial, organizational, talent, and supervisory support. However, four policies (P3, P17, P22, P40) scored between 0.5 and 0.75, while P22 scored only 0.25–-the lowest among all policies. This reflects a pattern where policies at higher administrative levels tend to provide more robust support mechanisms, while those at lower levels often lack concrete implementation details.
(3) Lowest-scoring dimensions. X1 Policy nature (0.65) reveals a predominance of guidance- and support-oriented content, with limited predictive or diagnostic elements–-a pattern that aligns with the developmental stage of the industry, where policy efforts are concentrated on industrial initiation rather than problem diagnosis and risk forecasting. X9 Issuing authority level (0.64) indicates that the policy system is predominantly composed of provincial and sub-provincial policies, with minimal national-level issuance, potentially compromising the authority required for cross-regional resource coordination and industrial synergy. The lowest mean score was recorded for X2 Policy timeliness (0.4), revealing that most policies concentrate on short- to medium-term planning cycles (3–5 years), with only two policies incorporating longer-term strategic elements extending beyond five years. This suggests that while annual work plans and three- to five-year action plans are well-developed, there is a widespread lack of sustained institutional arrangements for long-term industrial development.

Fig 8 presents the PMC surface plots for three representative policies: P35 (highest-scoring, PMC = 8.14), P22 (lowest-scoring, PMC = 5.42), and P3 (mean-representative, PMC = 6.3). The surface plots provide an intuitive visualization of the disparities in policy design completeness across these three tiers. The PMC surface plots for all 10 policies are provided in the supplementary materials (S2 Fig: excellent-rated policies; S3 Fig: good-rated policies).

**Fig 8:**
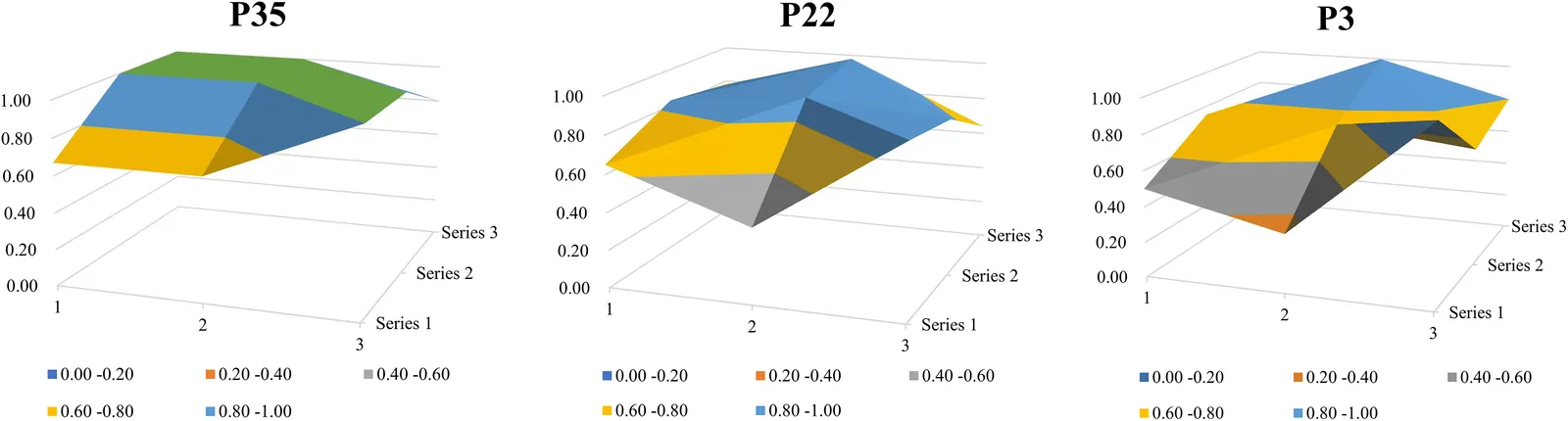
PMC surface plots for P35, P22, and P3.

The surface plot for P35 exhibits a large-area, high-altitude intact plateau with minimal concavity (concavity index = 0.86), reflecting comprehensive coverage across all nine dimensions. By contrast, the surface plot for P22 shows substantial concavities, particularly in X3 (policy content), X7 (support mechanisms), and X2 (policy timeliness), reflecting its predominantly principled orientation with limited concrete implementation mechanisms. The mean-representative policy P3, while falling within the “Good” category, exhibits concavities in X1 (policy nature), X2 (policy timeliness), and X7 (support mechanisms), suggesting that even average-performing policies share some of the structural deficiencies observed in lower-performing policies.

Among the excellent-rated policies, P35 achieves the highest score (PMC = 8.14), titled “Notice of Jiangxi Provincial People’s Government General Office on Issuing the Three-Year Action Plan (2023–2025) for Accelerating the High-Quality Development of the Selenium-Enriched Functional Agricultural Industry,” with full scores across policy content (X3), objectives (X4), recipients (X5), mode of action (X6), and support mechanisms (X7). Its minor deficiency lies in policy timeliness (X2 = 0.67), suggesting that even the best-performing policy could benefit from extending its strategic horizon beyond its current three-year action plan scope. At the opposite end, P22 represents the lowest-scoring policy (PMC = 5.42), titled “Notice of Guigang Municipal Bureau of Agriculture and Rural Affairs on Issuing the 2020 Guiding Opinions on the Development of Guigang’s Selenium-Enriched Industry,” with pronounced weaknesses in policy content (X3 = 0.4), support mechanisms (X7 = 0.25), and policy timeliness (X2 = 0.33), reflecting its predominantly principled orientation with limited concrete implementation mechanisms.

The concavity index, which measures the deviation of a policy from an ideal design, exhibits substantial variation across the sample, ranging from 0.86 (P35) to 3.58 (P22). A lower concavity index indicates a more balanced and internally consistent policy design. This variation suggests that while the overall policy system demonstrates good average coherence, individual policies differ markedly in their design completeness and internal consistency.

In summary, the assessment of the policy effectiveness dimension indicates that the current selenium-enriched policy system performs well in terms of content coverage, functional combination, and textual standardization. However, it exhibits notable deficiencies in long-term strategic planning and high-level systemic design, as reflected in the consistently low scores for policy timeliness (X2) and issuing authority level (X9). This reveals a structural characteristic whereby policies are “stronger in short-term operational measures and localized regulations, yet weaker in long-term vision and top-level coordination”.

## Conclusions

This study systematically and quantitatively analyzes 42 selenium-enriched policy texts issued in China between 2009 and 2025, delineating the policy response landscape for promoting the integrated development of specialized agriculture and health industries within the synergistic context of the “Healthy China” initiative and the rural revitalization strategy. This section summarizes the core findings, elucidates the research implications and outlines directions for future research.

### Key findings

China’s selenium-enriched policy system demonstrates strong capacity for rapid industrial formation through project-based implementation, yet reveals structural weaknesses in fostering long-term industrial sustainability.

First, the distribution of policy themes clearly points to a “dual-core driven” characteristic centered on industrial development and market cultivation. The LDA model results show that “Cultivation of distinctive selenium-enriched industries and brand support” and “Industrial integration and park development” dominate policy attention, indicating that policy resources are highly concentrated on value realization and industrial initiation. By contrast, systemic coordination, long-term risk prevention, and sustainable resource management receive substantially less weight. This imbalance suggests that while current policies effectively ignite industrial growth, they underinvest in building resilience against market and resource risks. Future policy design should incorporate the coordinated development of selenium-enriched land resources and industrial transformation into special plans, addressing soil protection, ecological risk control, and resource conservation zoning.

Second, policy instruments display a composite pattern of “supply-demand coexistence with internal functional differentiation.” Four structural features emerge: (1) supply- and demand-side instruments jointly dominate (74.45%), with environmental instruments playing a complementary regulatory role (25.55%); (2) within the supply side, hardware-oriented supports–-production base construction (11.56%) and technology R&D platforms (10.70%)–-prevail, while intangible inputs such as talent cultivation (2.99%), resource exploration (2.82%), and big-data services (2.71%) remain underutilized; (3) on the demand side, emphasis falls on industrial chain coordination and business entity cultivation (14.55%) rather than direct consumer engagement, with “Selenium & Wellness Tourism” models receiving the least attention (3.62%); and (4) environmental instruments exhibit a reactive orientation, prioritizing supervision and accountability over proactive resource conservation, as reflected in the low utilization of selenium resource protection measures (3.76%). This instrument structure suits rapid industrial initiation but may be less effective for fostering long-term resilience.

Finally, the assessment of policy effectiveness reveals a coexistence of “strong textual standardization and project operability, yet insufficient strategic foresight and top-level coordination”. The average PMC index of 6.9 indicates that policies exhibit good internal consistency in their design. However, the notably low score on the policy timeliness dimension (mean of 0.4) exposes a widespread short-term orientation: most policies cover only 3–5 years, with only two extending beyond five years. Concurrently, the issuing authority dimension (X9, mean 0.64) reveals that the policy system is overwhelmingly composed of provincial and sub-provincial instruments. A systematic search of central ministry databases yielded no national-level plans or guiding opinions specifically targeting the selenium-enriched industry. The system thus operates as “local pilot practices without national strategic coordination”–-a structure well-suited to rapid, context-sensitive responses, but potentially limiting the scalability of successful practices and cross-regional resource alignment. This suggests that the current policy system functions more effectively as a “project engineer” executing concrete development tasks than as a “strategic architect” capable of cross-cycle planning and top-level systemic design.

### Research implications

At the theoretical level, this study, by constructing and validating a replicable three-dimensional analytical framework, provides an operational “analytical lens” for deconstructing the complex governance issue of “specialized agricultural industry development policies.” It decomposes macro-level strategic narratives into three observable and measurable dimensions-policy themes, policy instruments, and policy design consistency–-not only systematically depicting the realistic landscape of China’s selenium-enriched industry policies but also revealing the underlying design logic and structural characteristics, such as the pragmatic prioritization of industrial and market initiation, the government-led path dependency in policy instrument selection, and the relative weakness in long-term planning and top-level coordination. This framework transcends case-specific descriptions of individual regions or industries, offering a broadly applicable methodological tool and empirical benchmark for public policy analysis, particularly for research on cross-cutting industrial policies encompassing multiple objectives such as health, economy, and environment. It establishes a foundation for dialogue and comparison in future cross-regional and cross-industry studies.

At the practical level, the findings provide policymakers with an evidence-based diagnostic tool and a clear optimization roadmap. Moving toward high-quality development requires a strategic elevation from the role of a “project engineer”–focused on rapid industrial formation through concrete initiatives–-to that of a “system architect” capable of designing resilient institutional frameworks for long-term industrial health.

Two priorities merit specific attention.

First, the persistently low policy timeliness scores (mean 0.4) reflect a systemic short-term orientation, attributable to both the industry’s early-stage uncertainty and the absence of national-level strategic guidance. Addressing this requires strengthening legislative planning at the national level–-establishing legally binding long-term targets, periodic review mechanisms, and stable resource allocation frameworks that transcend administrative cycles–-while simultaneously improving administrative planning at the local level through multi-year action plans, flexible implementation pathways, and incentive structures that reward sustained progress over short-term outputs.

Second, the instrument structure exhibits a distinct “production-side logic” that prioritizes firm cultivation and factor inputs over consumer engagement. Demand-side instruments are skewed toward industrial chain coordination (14.55%) rather than direct stimulation of health-conscious consumption. Notably, “Selenium & Wellness Tourism” business model cultivation accounts for just 3.62% of instruments–-the lowest share among demand-side subcategories. Its underdevelopment stems not only from insufficient demand-side investment, but also from fragmented local governance: the absence of cross-regional coordination mechanisms hinders unified tourism brands and interregional routes, driving homogeneous competition and preventing the formation of a national industrial IP and economies of scale. To address this, administrative planning should integrate health education, selenium literacy campaigns, and consumption subsidy pilots to align production with consumer demand. Legislative planning should codify quality standards, certification requirements, and traceability systems to build consumer trust. Concurrently, supply-side investment in selenium resource exploration and foundational data development–-currently at only 2.82%–-should be strengthened to support evidence-based industrial spatial planning.

### Future research directions

Building upon the findings of this study, several avenues for future research emerge as particularly promising.

First, the absence of national-level policies warrants closer investigation. No specialized national-level plans for the selenium-enriched industry were found in central ministry databases. This reflects China’s “local pilot-first” institutional logic for characteristic agricultural industries, wherein local governments experiment with policy design before central frameworks are formalized. Future research could examine whether this reflects a deliberate strategy of decentralized experimentation or an institutional gap that may constrain the industry’s long-term development. Comparative studies across regions with varying resource endowments could illuminate how local conditions shape policy responses and what central-local coordination mechanisms best support industry growth.

Second, the consistently low policy timeliness scores invite investigation into the causes of short-term orientation. Research could examine whether this pattern is attributable to the industry’s emerging nature–-where rapid market and technological changes make long-term planning inherently difficult–-or to broader institutional factors affecting policy horizon-setting across similar sectors.

Third, extending the analytical focus beyond policy texts toward implementation and outcomes represents a critical next step. Future research could examine enforcement effectiveness, resource allocation relative to stated priorities, stakeholder-perceived benefits, and measurable economic, social, and health outcomes. Complementary methodologies–-case tracking, stakeholder surveys, and quantitative performance assessment linking policy characteristics to industrial indicators–-would help bridge the gap between policy design and practice.

Fourth, the thematic and instrumental imbalances identified suggest the value of longitudinal analysis. While this study provides a cross-sectional snapshot (2009–2025), future research could trace how policy attention and instrument mixes evolve in response to changing industrial conditions, political priorities, and resource constraints–-examining how emerging issues transition from marginal to central positions and how tensions between resource protection and industrial development are balanced over time.

Fifth, the integrated LDA-content analysis-PMC framework offers a replicable methodological toolkit for comparative policy studies of other specialized agricultural industries, such as zinc-enriched products or organic agriculture. Such applications would test the framework’s generalizability across resource-based sectors and contribute to broader understanding of policy design for industries spanning agricultural production, health promotion, and economic development. Building upon controls for regional variations and resource endowments, subsequent investigations could further refine the framework’s applicability and generate more broadly generalizable policy design insights.

Sixth, cross-national comparative analysis offers a concrete research direction. Finland’s mandatory selenium fertilization program and the United States’ FDA regulatory approach represent governance models distinct from China’s locally driven pilot practices. Future research could compare policy instruments and effectiveness outcomes across these three countries, applying the three-dimensional framework developed in this study to test its generalizability.

By pursuing these directions, future research can build upon the foundations established in this study–-shifting the analytical focus from diagnosing the structure of policy texts to understanding the conditions under which policy design translates into tangible outcomes, thereby contributing to more effective governance of specialized agriculture and health-promoting industries.

## Supporting information

S1 Table Complete list of 42 selenium-enriched policy documents (2009–2025): issuing authority, release date and policy grade. This table presents the 42 selenium-enriched policies analyzed in this study, including their title, issuing authority, and issuance date.
(PDF)

S2 Table Topic coherence scores for the LDA model with different numbers of topics (*K* = 1–10). (PDF)

S3 Table PMC scoring sheets: X1-X4. (PDF)

S4 Table PMC scoring sheets: X5-X9. (PDF)

S1 Fig pyLDAvis visualization of the six-topic LDA model. A: Theme 1–Cultivation of distinctive selenium-enriched industries and brand support; B: Theme 2–Integration of selenium-enriched industries and park development; C: Theme 3–Industrial chain coordination and talent support; D: Theme 4–Planning and financial guarantees; E: Theme 5–Quality control and technology promotion; F: Theme 6–Specialized selenium-enriched cultivation and ecological base construction. The top 30 characteristic terms for each topic are displayed within each corresponding bubble plot.
(ZIP)

S2 Fig PMC surface plot of excellent-rated policies. A: PMC surface plot for P6; B: PMC surface plot for P11; C: PMC surface plot for P26; D: PMC surface plot for P35.
(ZIP)

S3 Fig PMC surface plot of good-rated policies. A: PMC surface plot for P3; B: PMC surface plot for P17; C: PMC surface plot for P22; D: PMC surface plot for P23; E: PMC surface plot for P40; F: PMC surface plot for P42.
(ZIP)
