# Supplementary material for: Quantitative analysis of China’s selenium-enriched policy texts: Based on a three-dimensional framework of “theme–instrument–effectiveness”
Source: PLoS One. 2026 Oct 13;21(10):e0360381. doi: 10.1371/journal.pone.0360381 (PMC13660983; doi:10.1371/journal.pone.0360381)
Supplement: Supplementary file 1 [file pone.0360381.s001.pdf]

Supporting information

S1 Table. Complete list of 42 selenium-enriched policy documents (2009–2025): issuing authority, release date and policy grade.

This table presents the 42 selenium-enriched policies analyzed in this study, including their title, issuing authority, and issuance date.

No.	Policy title	Issuing authority	Issued date
P1	Opinions of Ankang Municipal People's Government on Promoting the Development of the Selenium-Enriched Food Industry	Ankang Municipal People's Government	July 1, 2009
P2	Opinions of Jiangjin District People's Government, Chongqing Municipality on Accelerating the Development of Distinctive Selenium-Enriched Industries	Jiangjin District People's Government, Chongqing	October 19, 2012
P3	Opinions of Fujian Provincial Department of Agriculture on Accelerating the Development of the Selenium-Enriched Agricultural Industry	Fujian Provincial Department of Agriculture	October 20, 2016
P4	Notice on Issuing the Special-Action Plan for Further Supporting the Development of Ankang's Selenium-Enriched Food Industry (2016–2020)	Shaanxi Provincial Department of Industry and Information Technology	November 28, 2016
P5	Notice of Nanning Municipal Agricultural Committee on Issuing the 2017 Implementation Plan for Selenium-Enriched Agricultural Development	Nanning Municipal Agricultural Committee	March 21, 2017
P6	Notice of Ankang Municipal People's Government General Office on Issuing the Action Plan for the Development of Ankang's Selenium-Enriched Industry (2017–2020)	Ankang Municipal People's Government General Office	April 18, 2017
P7	Implementation Opinions of Laibin Municipal People's Government General Office on Promoting the Development of the Selenium-Enriched Industry	Laibin Municipal People's Government General Office	May 18, 2017
P8	Opinions of Ziyang County Committee of the CPC and Ziyang County People's Government on Accelerating the Development of the Selenium-Enriched Ecological Industrial Park	Ziyang County People's Government	December 13, 2017
P9	Notice of Guigang Municipal People's Government General Office on Issuing the Development Plan for Guigang's Selenium-Enriched Industry (2018–2022)	Guigang Municipal People's Government General Office	May 23, 2018
P10	Notice of Zhongwei Municipal People's Government General Office on Issuing the Promotion Plan for the Selenium-Enriched Edible Fungus Industry	Zhongwei Municipal People's Government General Office	May 25, 2018

No.	Policy title	Issuing authority	Issued date
P11	Notice of the Department of Agriculture of the Autonomous Region on Issuing the 2018 Work Plan for the Development of the Selenium-Enriched Agricultural Industry	Department of Agriculture of Guangxi Zhuang Autonomous Region	May 29, 2018
P12	Notice of Ankang Municipal People's Government General Office on Issuing the Brand Integration Plan for Ankang Selenium-Enriched Tea	Ankang Municipal People's Government General Office	August 19, 2018
P13	Guiding Opinions of Ganzhou Municipal People's Government General Office on Fully Utilizing Selenium-Enriched Land Resources to Promote the Development of Selenium-Enriched Agricultural Products	Ganzhou Municipal People's Government General Office	September 2, 2018
P14	Notice of Yichun Municipal People's Government on Issuing Implementation Opinions on Further Accelerating the Development of the Selenium-Enriched Industry	Yichun Municipal People's Government	December 20, 2018
P15	2019 Implementation Plan for the Development of the Selenium-Enriched Industry in Zhongwei City	Zhongwei Municipal People's Government General Office	February 28, 2019
P16	Notice of the Department of Agriculture and Rural Affairs of the Autonomous Region on Issuing the 2019 Work Plan for Brand Building of Guangxi's Selenium-Enriched Agriculture	Department of Agriculture and Rural Affairs of Guangxi Zhuang Autonomous Region	March 21, 2019
P17	Notice of Jiangjin District People's Government General Office, Chongqing Municipality on Issuing the Key Work Points for the Development of Jiangjin District's Selenium-Enriched Industry in 2019	Jiangjin District People's Government General Office, Chongqing	April 15, 2019
P18	Notice of Chongzuo Municipal Bureau of Agriculture and Rural Affairs on Issuing the 2019 Implementation Plan for Brand Building of Chongzuo's Selenium-Enriched Agriculture	Chongzuo Municipal Bureau of Agriculture and Rural Affairs	May 10, 2019
P19	Notice of Ankang Municipal People's Government General Office on Issuing the 2019 Work Plan and Assessment Measures for the Development of the Selenium-Enriched Industry	Ankang Municipal People's Government General Office	August 30, 2019
P20	Guiding Opinions of Jiangxi Provincial Department of Agriculture and Rural Affairs on Accelerating the High-Quality Development of Selenium-Enriched Agriculture	Jiangxi Provincial Department of Agriculture and Rural Affairs	September 27, 2019
P21	Several Measures on Accelerating the High-Quality and Leapfrog Development of the Selenium-Enriched Industry in the City	Yichun Municipal Bureau of Agriculture and Rural Affairs	December 30, 2019
P22	Notice of Guigang Municipal Bureau of Agriculture and Rural Affairs on Issuing the 2020 Guiding Opinions on the Development of Guigang's Selenium-Enriched Industry	Guigang Municipal Bureau of Agriculture and Rural Affairs	March 13, 2020

No.	Policy title	Issuing authority	Issued date
P23	Notice of Jiangxi Provincial Department of Agriculture and Rural Affairs General Office on Issuing the Key Work Points for the Development of the Provincial Selenium-Enriched Agricultural Industry in 2020	Jiangxi Provincial Department of Agriculture and Rural Affairs General Office	March 25, 2020
P24	Notice of Ankang Municipal People's Government General Office on Issuing the 2020 Work Plan and Assessment Measures for the Development of the Selenium-Enriched Industry	Ankang Municipal People's Government General Office	March 26, 2020
P25	Notice of Ankang Municipal People's Government General Office on Issuing the Five-Year Enhancement Action Plan for Ankang's Five Major Distinctive Selenium-Enriched Agricultural Industries (2021–2025)	Ankang Municipal People's Government General Office	March 19, 2021
P26	Notice on Issuing the Five-Year Action Plan for the Selenium-Enriched Agricultural Industry of Lianzhou City	Lianzhou Municipal People's Government General Office	June 25, 2021
P27	Notice of Ankang Municipal People's Government General Office on Issuing the Three-Year Action Plan for the High-Quality Development of Ankang's Whole Selenium-Enriched Industry Chain (2021–2023)	Ankang Municipal People's Government General Office	July 22, 2021
P28	Notice of Yudu County People's Government General Office on Issuing the Implementation Opinions on the Development of Yudu County's Selenium-Enriched Agricultural Industry (Trial)	Yudu County People's Government General Office	July 30, 2021
P29	Notice of Shicheng County People's Government General Office on Issuing the 2021 Work Plan for the Development of Shicheng County's Selenium-Enriched Agricultural Industry	Shicheng County People's Government General Office	September 22, 2021
P30	Notice of Ankang Municipal People's Government on Issuing the Development Plan for Ankang's Selenium-Enriched Industry during the "14th Five-Year Plan" Period	Ankang Municipal People's Government	September 29, 2021
P31	Notice of Ankang Municipal People's Government General Office on Issuing the Implementation Plan for the Whole-Process Quality Control Pilot City for National Famous, Special, Excellent, and New High-Quality (Selenium-Enriched) Agricultural Products (2021–2023)	Ankang Municipal People's Government General Office	January 13, 2022
P32	Notice of the Department of Agriculture and Rural Affairs of the Autonomous Region General Office on Issuing the 2022 Work Plan for Promoting the High-Quality Development of Guangxi's Selenium-Enriched Agriculture	Department of Agriculture and Rural Affairs of Guangxi Zhuang Autonomous Region General Office	April 29, 2022

No.	Policy title	Issuing authority	Issued date
P33	Notice of Yudu County People's Government General Office on Issuing the Key Work Points for the Development of Yudu County's Selenium-Enriched Industry in 2022	Yudu County People's Government General Office	May 26, 2022
P34	Notice on Issuing the Implementation Plan for Accelerating the High-Quality Development of Pingxiang City's Selenium-Enriched Agriculture (2022–2025)	Pingxiang Municipal Bureau of Agriculture and Rural Affairs	August 9, 2022
P35	Notice of Jiangxi Provincial People's Government General Office on Issuing the Three-Year Action Plan (2023–2025) for Accelerating the High-Quality Development of the Selenium-Enriched Functional Agricultural Industry	Jiangxi Provincial People's Government General Office	September 1, 2022
P36	Notice of the Provincial Development and Reform Commission and the Provincial Geological Bureau on Issuing the Development Plan for Hubei Province's Selenium-Enriched Industry (2021–2025)	Hubei Provincial Development and Reform Commission; Hubei Provincial Geological Bureau	October 10, 2022
P37	Notice of Jiangxi Provincial Department of Agriculture and Rural Affairs on Issuing the Development Plan for Jiangxi Province's Selenium-Enriched Functional Agricultural Industry (2023–2025)	Jiangxi Provincial Department of Agriculture and Rural Affairs	December 14, 2022
P38	Notice of Jiangxi Provincial Department of Agriculture and Rural Affairs on Issuing Jiangxi Province's "14th Five-Year Plan" for Rural Industrial Development	Jiangxi Provincial Department of Agriculture and Rural Affairs	March 16, 2023
P39	Notice of Fenggang County People's Government on Issuing the Three-Year Action Implementation Opinions on the Development of Fenggang County's Zinc-Selenium Functional Agricultural Industry	Fenggang County People's Government	December 31, 2024
P40	Notice of Ping'an District People's Government General Office, Haidong City on Issuing the 2025 Implementation Plan for the Development of the Selenium-Enriched Industry in Ping'an District	Ping'an District People's Government General Office, Haidong City	June 16, 2025
P41	Notice on Issuing the Guideline for Building the Standard System of Shaanxi Province's Selenium-Enriched Industry (2025 Edition)	Shaanxi Provincial Administration for Market Regulation; Shaanxi Provincial Department of Industry and Information Technology	November 13, 2025
P42	Regulations of Yangquan City on Promoting the Development of the Selenium-Enriched Agricultural Industry	Yangquan Municipal People's Congress Standing Committee	November 26, 2025
