# Supplementary material for: Quantitative analysis of China’s selenium-enriched policy texts: Based on a three-dimensional framework of “theme–instrument–effectiveness”
Source: PLoS One. 2026 Oct 13;21(10):e0360381. doi: 10.1371/journal.pone.0360381 (PMC13660983; doi:10.1371/journal.pone.0360381)
Supplement: Supplementary file 2 [file pone.0360381.s002.pdf]

Supporting information

S2 Table. Topic coherence scores for the LDA model with different numbers of topics ($K = 1$–10).

Number of topics (K)	Coherence score
1	0.2949
2	0.3295
3	0.2823
4	0.3290
5	0.3339
6	**0.3406**
7	0.3066
8	0.3245
9	0.3035
10	0.3103

Higher coherence scores indicate stronger semantic consistency within topics. The maximum value at $K = 6$ represents the optimal number of latent topics.
