# Supplementary material for: Quantitative analysis of China’s selenium-enriched policy texts: Based on a three-dimensional framework of “theme–instrument–effectiveness”
Source: PLoS One. 2026 Oct 13;21(10):e0360381. doi: 10.1371/journal.pone.0360381 (PMC13660983; doi:10.1371/journal.pone.0360381)
Supplement: Supplementary file 3 [file pone.0360381.s003.pdf]

# Supporting information

**S3 Table. PMC scoring sheets: X1-X4.**

| Policy | X1 Policy nature | | | | | | X2 Timeliness | | | X3 Policy content | | | | | X4 Objectives | | | |
|---|---|---|---|---|---|---|---|---|---|---|---|---|---|---|---|---|---|---|
| | 1:1 | 1:2 | 1:3 | 1:4 | 1:5 | 1:6 | 2:1 | 2:2 | 2:3 | 3:1 | 3:2 | 3:3 | 3:4 | 3:5 | 4:1 | 4:2 | 4:3 | 4:4 |
| P3 | 0 | 1 | 0 | 1 | 1 | 0 | 0 | 1 | 0 | 1 | 1 | 1 | 1 | 1 | 1 | 1 | 1 | 0 |
| P6 | 0 | 1 | 1 | 1 | 1 | 0 | 1 | 0 | 0 | 1 | 1 | 1 | 1 | 1 | 1 | 1 | 1 | 1 |
| P11 | 0 | 1 | 1 | 1 | 1 | 0 | 1 | 0 | 0 | 1 | 1 | 1 | 1 | 1 | 1 | 1 | 1 | 0 |
| P17 | 1 | 1 | 1 | 1 | 1 | 1 | 1 | 0 | 0 | 1 | 1 | 1 | 1 | 1 | 1 | 1 | 1 | 1 |
| P22 | 0 | 1 | 1 | 1 | 0 | 1 | 1 | 0 | 0 | 0 | 1 | 0 | 0 | 1 | 1 | 1 | 1 | 0 |
| P23 | 0 | 1 | 0 | 1 | 1 | 0 | 1 | 0 | 0 | 1 | 1 | 1 | 0 | 1 | 1 | 1 | 1 | 0 |
| P26 | 0 | 1 | 0 | 1 | 1 | 0 | 1 | 1 | 0 | 1 | 1 | 1 | 1 | 1 | 1 | 1 | 1 | 1 |
| P35 | 0 | 1 | 1 | 1 | 1 | 0 | 1 | 1 | 0 | 1 | 1 | 1 | 1 | 1 | 1 | 1 | 1 | 1 |
| P40 | 0 | 1 | 0 | 1 | 1 | 0 | 1 | 0 | 0 | 1 | 1 | 1 | 0 | 1 | 1 | 1 | 1 | 0 |
| P42 | 0 | 1 | 1 | 1 | 1 | 1 | 0 | 0 | 1 | 1 | 1 | 1 | 1 | 1 | 0 | 0 | 1 | 1 |
