# Supplementary material for: Quantitative analysis of China’s selenium-enriched policy texts: Based on a three-dimensional framework of “theme–instrument–effectiveness”
Source: PLoS One. 2026 Oct 13;21(10):e0360381. doi: 10.1371/journal.pone.0360381 (PMC13660983; doi:10.1371/journal.pone.0360381)
Supplement: Supplementary file 4 [file pone.0360381.s004.pdf]

Supporting information

S4 Table. PMC scoring sheets: X5-X9.

Policy	X5 Recipients				X6 Action			X7 Support				X8 Evaluation				X9
	5:1	5:2	5:3	5:4	6:1	6:2	6:3	7:1	7:2	7:3	7:4	8:1	8:2	8:3	8:4	
P3	1	1	1	0	0	1	1	1	0	1	0	1	1	1	1	0.8
P6	1	1	1	1	1	1	1	1	1	1	1	1	1	1	1	0.6
P11	1	1	1	1	1	1	1	1	1	1	1	1	1	1	1	0.8
P17	1	1	1	0	1	1	1	1	0	1	0	1	1	1	1	0.4
P22	1	0	1	1	1	1	0	0	0	0	1	1	1	1	1	0.6
P23	1	1	1	1	0	1	1	1	1	1	0	1	1	1	1	0.8
P26	1	1	1	1	0	1	1	1	1	1	1	1	1	1	1	0.6
P35	1	1	1	1	1	1	1	1	1	1	1	1	1	1	1	0.8
P40	1	1	1	1	0	1	1	1	1	1	0	1	1	1	1	0.4
P42	1	1	1	0	1	1	1	1	1	1	1	1	0	1	1	0.6

Note: X9 weights: national=1.0, provincial=0.8, municipal=0.6, county=0.4.
